# Epoxidized graphene grid for highly efficient high-resolution cryoEM structural analysis

**DOI:** 10.1038/s41598-023-29396-0

**Published:** 2023-02-08

**Authors:** Junso Fujita, Fumiaki Makino, Haruyasu Asahara, Maiko Moriguchi, Shota Kumano, Itsuki Anzai, Jun-ichi Kishikawa, Yoshiharu Matsuura, Takayuki Kato, Keiichi Namba, Tsuyoshi Inoue

**Affiliations:** 1 Graduate School of Frontier Biosciences, Osaka University, 1-3 Yamadaoka, Suita, Osaka 565-0871 Japan; 2 JEOL YOKOGUSHI Research Alliance Laboratories, Osaka University, 1-3 Yamadaoka, Suita, Osaka 565-0871 Japan; 3 Graduate School of Pharmaceutical Sciences, Osaka University, 1-6 Yamadaoka, Suita, Osaka 565-0871 Japan; 4 JEOL Ltd, 3-2-1 Musashino, Akishima, Tokyo 196-8558 Japan; 5 Open and Transdisciplinary Research Initiatives, Osaka University, 2-8 Yamadaoka, Suita, Osaka 565-0871 Japan; 6 Department of Molecular Virology, Research Institute for Microbial Diseases, Osaka University, 3-1 Yamadaoka, Suita, Osaka 565-0871 Japan; 7 Institute for Protein Research, Osaka University, 3-2 Yamadaoka, Suita, Osaka 565-0871 Japan; 8 Department of Molecular Biosciences, Kyoto Sangyo University, Motoyama Kamigamo, Kita-ku, Kyoto 603-8555 Japan; 9 Center for Infectious Disease Education and Research, Osaka University, 2-8 Yamadaoka, Suita, Osaka 565-0871 Japan; 10 Laboratory of Virus Control, Research Institute for Microbial Diseases, Osaka University, 3-1 Yamadaoka, Suita, Osaka 565-0871 Japan; 11 RIKEN Center for Biosystems Dynamics Research and SPring-8 Center, 1-3 Yamadaoka, Suita, Osaka 565-0871 Japan; 12 dotAqua Inc., 2-1 Yamadaoka, Suita, Osaka Japan

**Keywords:** Photochemistry, Electron microscopy, Cryoelectron microscopy, Structural biology, Chemistry

## Abstract

Functionalization of graphene is one of the most important fundamental technologies in a wide variety of fields including industry and biochemistry. We have successfully achieved a novel oxidative modification of graphene using photoactivated ClO_2_^·^ as a mild oxidant and confirmed the oxidized graphene grid is storable with its functionality for at least three months under N_2_ atmosphere. Subsequent chemical functionalization enabled us to develop an epoxidized graphene grid (EG-grid™), which effectively adsorbs protein particles for electron cryomicroscopy (cryoEM) image analysis. The EG-grid dramatically improved the particle density and orientation distribution. The density maps of GroEL and glyceraldehyde 3-phosphate dehydrogenase (GAPDH) were reconstructed at 1.99 and 2.16 Å resolution from only 504 and 241 micrographs, respectively. A sample solution of 0.1 mg ml^−1^ was sufficient to reconstruct a 3.10 Å resolution map of SARS-CoV-2 spike protein from 1163 micrographs. The map resolutions of β-galactosidase and apoferritin easily reached 1.81 Å and 1.29 Å resolution, respectively, indicating its atomic-resolution imaging capability. Thus, the EG-grid will be an extremely powerful tool for highly efficient high-resolution cryoEM structural analysis of biological macromolecules.

## Introduction

Despite the recent improvements in hardware and software for electron cryomicroscopy (cryoEM) single particle image analysis for high-throughput and high-resolution structure determination of biological macromolecules, specimen grid preparation remains a bottleneck due to various factors. In single particle analysis (SPA), protein particles must be well dispersed in aqueous solution to be flash-frozen and embedded in thin vitreous ice film formed within micron-sized holes on a few 10s nm thick carbon film laid on a metal grid substrate^1^. Automated vitrification devices have been developed^2–5^, and various efforts have been made to produce good grids with well-dispersed particles and without much sample loss by denaturation^4–8^. In general, proteins often localize to the air–water interface and denature, resulting in poor particle density and preferred orientation and thereby making high-resolution 3D reconstruction inefficient^9^. Due to these problems, optimizing the conditions for cryoEM grid preparation requires much time and effort^10,11^. To overcome these problems, thin support films, such as graphene, have been used to change the physical properties of grid surface^12–14^, and various types of functionalization have been achieved: Nα, Nα-dicarboxymethyllysine (NTA)^15,16^; SpyTag/SpyCatcher^17^; amino-polyethylene glycol (PEG) linkers^18^; as well as various organic compounds^19^. Although synthesis of graphene oxide is the first important step for graphene functionalization, a limited number of methods have been available: modified Hummers’ methods^20,21^ (chemical wet process); conventional glow discharge; and chemical modification by plasma treatment^22^ (physical dry process). However, these methods suffer from problems such as undesirable breakage of the graphene framework (C–C bonds) and low selectivity of reaction points between the basal plane and the edges of graphene due to the harsh reaction conditions. It would be more useful if we can oxidize graphene in a milder condition and introduce appropriate functional groups into the basal plane of graphene selectively.

We have recently discovered a useful chemical reaction using photoactivated ClO_2_^·^ gas as a oxidant; it can convert a gaseous fuels of methane into liquid fuels of methanol and formic acid without producing CO_2_^23^ and can also make surface oxidation modification of polymers, such as polypropylene^24^. We applied this oxidation reaction to introduce hydroxy groups into the basal plane of graphene surface on the EM grid and confirmed the oxidized graphene grid is storable with its functionality for at least three months under N_2_ atmosphere. This is the first effective method as a chemical dry process for oxidation of graphene on substrate. We then successfully introduced epoxy groups by epichlorohydrin (ECH) treatment, which has been used for functionalization of graphene oxide^25,26^. The resulting epoxidized graphene grid (EG-grid™) effectively adsorbed proteins so that they stayed on the surface even after washing with buffer. This was confirmed using electron cryotomography (cryo-ET), which showed that the protein particles were located close to the graphene surface and away from the air–water interface to avoid their denaturation.

The EG-grid allows protein particles to be immobilized on graphene in a variety of orientation, not only making the adjustment of sample protein concentration less needed but also solving the problem of preferred orientation caused by adsorption to the air–water interface. In its application tests using standard proteins, such as GroEL and β-galactosidase, as well as difficult proteins with preferred orientation, such as glyceraldehyde 3-phosphate dehydrogenase (GAPDH) and SARS-CoV-2 spike protein, the numbers of cryoEM images needed for high-resolution reconstruction were much fewer than the cases where standard holey carbon grids were used. The resolution of apoferritin easily reached 1.29 Å resolution, also indicating its atomic-resolution imaging capability. We believe this EG-grid will become a powerful tool for accelerating high-resolution cryoEM structure determination.

## Results

### Characterization of ClO_2_^·^ oxidized grapheme

**Figure 1:**
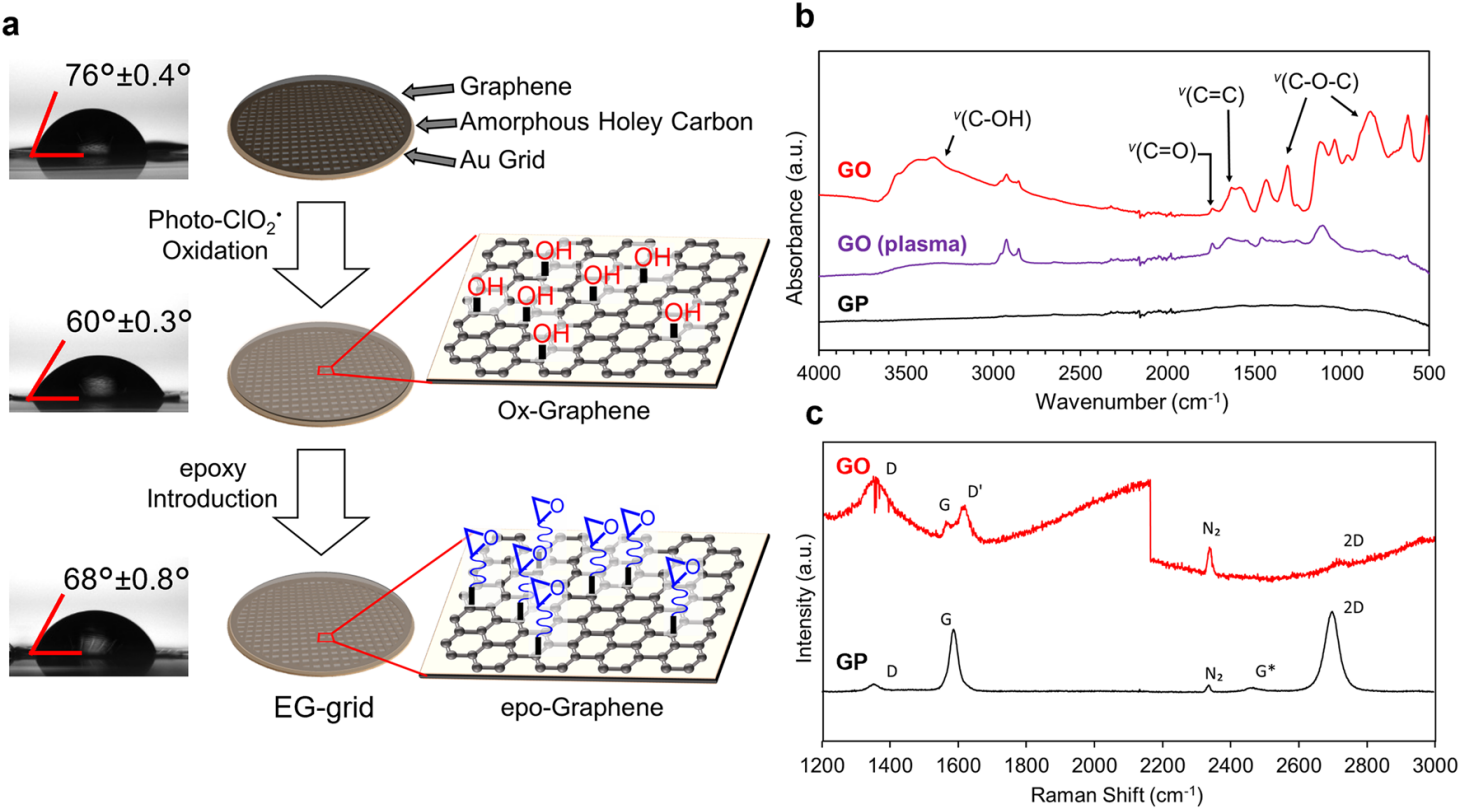
Fabrication process of the EG-grid and characterization of the graphene surface. (**a**) Water contact angle measurement and schematic illustration of EG-grid preparation by oxidation and functionalization of graphene on the Quantifoil Au grid. Data are obtained as the mean of three independent experiments with standard deviation. (**b**) IR spectra of pristine graphene (GP: black line), plasma treated graphene (GO (plasma): purple line), and oxidized graphene after 10 min ClO_2_^·^ treatment (GO: red line) on Cu foil. (**c**) Raman spectra of GP (black line) and GO (red line) on silicon wafer.

The EG-grid is produced in three steps as shown in a schematic illustration of the fabrication process (Fig. 1a). First, a CVD graphene layer is put on a Quantifoil Au grid as previously reported^13^ with some modification. Then, the surface of graphene is oxidized by photoactivated ClO_2_^·^ treatment for 10 min to introduce hydroxy groups (see “Methods” for detail and Supplementary Movie 1). The following reaction with ECH in the third step introduces epoxy groups on the surface of graphene. The hydrophilicity of graphene grids was evaluated by the water contact angle (WCA) measurement. The WCA of graphene on the Au grid decreased from 76° ± 0.4° to 60° ± 0.3° by the ClO_2_^·^ oxidation treatment (Fig. 1a, left panels). The introduction of epoxy groups slightly decreased the hydrophilicity (68° ± 0.8°).

To confirm the oxidation state of the graphene surface, we performed infrared (IR) and Raman spectral measurements and analyses. While pristine graphene (GP) was IR inactive as shown in Fig. 1b, graphene oxidized by ClO_2_^·^ (GO) showed several characteristic peaks such as those at 3400 cm^−1^, ^*v*^(C–OH); 1620 cm^−1^, ^*v*^(C=C); and 1300 cm^−1^, 850 cm^−1^, ^*v*^(C–O–C), suggesting that oxygen-containing functional groups were successfully introduced to the graphene surface. Contrary to this, the plasma-treated graphene showed a peak at 1740 cm^−1^ corresponds to the C=O bond and almost no C–O bond derived peaks. The Raman spectra of GP and GO are shown in Fig. 1c, in which GP showed two main peaks: G (1580 cm^−1^) and 2D (2690 cm^−1^), and their intensity ratio (*I*_2D_/*I*_G_) was about 1.8, proving the high quality of single layer graphene. After oxidation, the D and D’ peaks appeared whereas the 2D peak nearly diminished. These features of the Raman spectra indicated the formation of sp^3^-carbon by the introduction of functional groups to graphene basal plane.

To investigate the effect of oxidation on the chemical state of the graphene surface more comprehensively, the chemical compositions of the functional groups were determined by X-ray photoelectron spectroscopy (XPS) measurement. Graphene was transferred onto a silicon wafer and then oxidized either by plasma or photoactivated ClO_2_^·^ treatment. After plasma treatment, the composition ratios of the C–O groups (corresponding to the peak at 286.0 eV) and the C=O groups (corresponding to the peak at 287.8 eV) were 22.5% and 29.2%, respectively (Supplementary Fig. 1a). On the other hand, in the ClO_2_^·^-treated graphene, they were 28.4% (C–O) and 8.5% (C=O), respectively (Supplementary Fig. 1b). Generally, the increase of C=O ratio means the cleavage of the C=C bond of graphene. The increase of C–O ratio indicated that the hydroxy groups were introduced dominantly to the basal plane in the ClO_2_^·^-treated graphene rather than the cleavage of the C=C bond in the plasma-treated graphene, in agreement with the IR measurement.

To confirm the introduction of epoxy groups, we used 1H,1H-undecafluorohexylamine (UFHA) because the amino groups in this compound should react with the epoxy groups on the graphene surface and fluorine peaks can be clearly observed in XPS measurement (Supplementary Fig. 1c). Only carbon and oxygen were detected with non-oxidized graphene (black line). No fluorine was detected with the UFHA-treated oxidized graphene and ECH-/UFHA-treated graphene (red and green lines), while a clear fluorine peak appeared with the UFHA-treated EG-grid (purple line). These results indicated that UFHA was successfully introduced on the graphene surface and that the epoxy groups enabled further chemical modification of the EG-grid.

We observed the EG-grid using an electron cryomicroscope (cryoTEM) equipped with a cold field emission gun and an Ω-type in-column energy filter (JEM-Z300FSC (CRYO ARM™ 300), JEOL) operated at 300 kV and recorded images on a K3 direct electron detector (Gatan, Inc.). Most part (> 90–95%) of the EG-grid was covered with graphene (Supplementary Fig. 2a) while we sometimes found graphene-detached area by recognizing rolled-up edges of graphene (Supplementary Fig. 2b). The surface of graphene within the holes was quite clean (Supplementary Fig. 2c,d), and the existence of suspended graphene was confirmed by electron diffraction patterns (Supplementary Fig. 2e). To estimate the graphene damage caused by the oxidation and epoxidation, we recorded diffraction images of suspended graphene in 5 holes of three different types of grids: non-oxidized graphene grid (Gra); ClO_2_^·^ oxidized graphene grid (Ox-Gra); and EG-grid, and compared the peak intensities of the diffraction spots. We counted the maximum values of six equivalent diffraction peaks from the {${0}\overline{1}{\text{10}}$} and {${1}\overline{2}{\text{10}}$} lattice planes for each image and calculated the mean values. All the three grids showed very similar diffraction peak intensities from both of the lattice planes, and the intensity ratios before and after oxidation/epoxidation were ~ 1.0 (Supplementary Fig. 2f.), indicating that little damage was caused to graphene on the EG-grid during ClO_2_^·^ oxidation, epoxidation, and vitrification.

### Protein binding activity of the EG-grid

To investigate the protein binding activity of the EG-grid, we used GroEL from *Escherichia coli* (Molecular weight (*M*_w_) ~ 800 kDa) as a benchmark protein because the resolution of the cryoEM 3D reconstruction of this protein has been limited to 3.26 Å possibly by limited number of particles due to elimination of damaged particle images and by preferred orientation towards its end-on views^27^. A 3 μl solution of GroEL at a protein concentration of 0.3 mg ml^−1^ was applied on an EG-grid, and the protein solution was blotted after 5 min incubation. The grid was washed three times with 5 μl of buffer each, and then the grid was negatively stained with uranyl acetate. Many GroEL particles remained on the EG-grid and were packed densely (Supplementary Fig. 3a). We also prepared a grid in the same way except skipping the epoxidation process by ECH and observed no or very small number of GroEL particles (Supplementary Fig. 3b), which indicates that the EG-grid strongly adsorbed the protein particles.

### CryoEM image analysis of GroEL on the EG-grid

**Figure 2:**
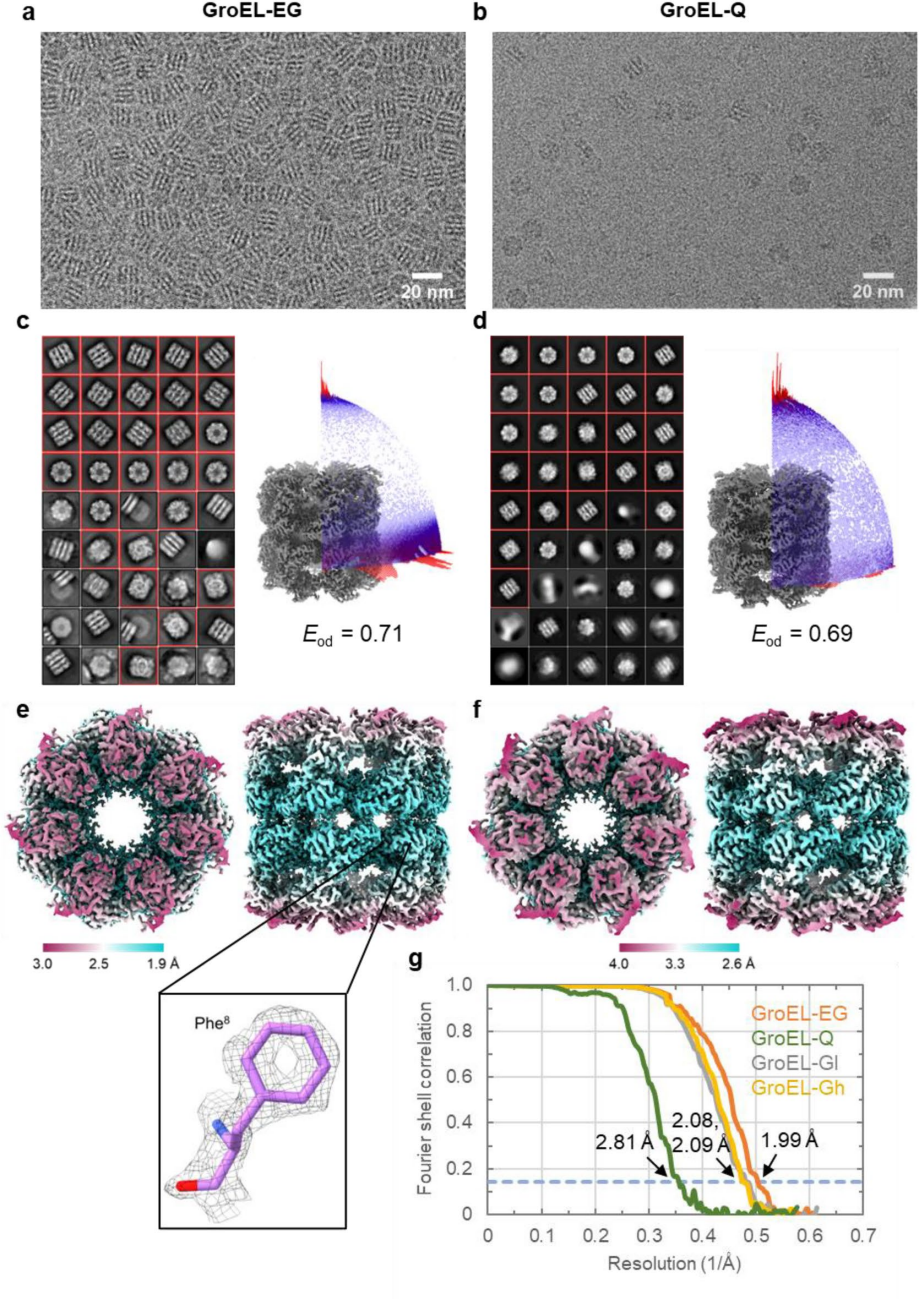
CryoEM image analyses of GroEL on the EG-grid and holey carbon grid. (**a** and **b**) Typical cryoEM images of GroEL on the EG-grid (GroEL-EG) (**a**) and on the glow discharged Quantifoil grid (GroEL-Q) (**b**). (**c** and **d**) 2D class averages from the GroEL-EG (**c**) and GroEL-Q (**d**) dataset. The class averages are aligned in descending order of particle numbers from left to right and top to bottom. The classes selected for the following analysis are indicated with red boxes. Right panel shows angular distribution of particles used in the final refinement for each dataset. Final 3D maps are shown for reference. The efficiencies (*E*_od_) calculated with cryoEF are also shown. (**e** and **f**) Two orthogonal views of the final 3D map from the GroEL-EG (**e**) and GroEL-Q (**f**) dataset: top view, left panel; and side view, right panel. The local resolution distribution is colored as in the color bar. The inset shows the density map and the fitted model (PDB: 1SX3) around Phe^8^. (**g**) The FSC curves for the final maps of the GroEL-EG and GroEL-Q dataset. The dashed blue line indicates the FSC = 0.143 criterion.

For cryoEM image analysis of GroEL, 3 μl of 1.5 mg ml^−1^ GroEL solution was loaded onto the EG-grid and allowed to stand for 5 min at room temperature, followed by blotting and plunge freezing using Vitrobot™ (Thermo Fisher Scientific). A short blotting time around 1.0–1.5 s usually worked well for getting appropriate ice thickness on the EG-grid. We observed the grid and collected a dataset using CRYO ARM™ 300 operated at 300 kV (referred to as GroEL-EG). For comparison with a conventional grid preparation method, we also prepared a cryoEM grid using a Quantifoil grid and collected a dataset under the same EM imaging condition (referred to as GroEL-Q). We collected around 500 micrographs in both cases. The micrographs of GroEL-EG showed much more densely packed GroEL particles with more side view orientations than GroEL-Q (Fig. 2a,b). Having more side view orientations is favorable for high-resolution 3D reconstruction due to the cylindrical structure of GroEL. The 2D class averages and orientation distributions of the particles used for final reconstructions also indicated the side-view preference of GroEL-EG (Fig. 2c,d). In the GroEL-EG dataset, 158,485 particles picked from 500 micrographs contributed to the final reconstruction, and the overall map resolution reached 1.99 Å, which is much higher than the previous study^28^, with a sufficient quality to show the holes of aromatic rings of Phe residues (Fig. 2e; Supplementary Table 1). In contrast, for the GroEL-Q dataset, only 20,061 particles obtained from 553 micrographs were used for the final reconstruction due to the much lower particle density, and the overall map resolution was limited to 2.81 Å (Fig. 2f–g; Supplementary Table 1).

To confirm whether the differences in the particle density and the orientation distribution between these grids were caused by the chemical modification of graphene on the EG-grid, we additionally collected two datasets from two cryoEM grids prepared by using Quantifoil grids covered with graphene. We hydrophilized the graphene surface by glow discharge, but since this treatment tends to damage the graphene layer and it is difficult to reproduce a similar level of hydrophilicity on the graphene surface, we treated the two graphene grids in two separate sessions to see the reproducibility of treatment. Micrographs imaged on one grid (dataset referred to as GroEL-Gl) showed a much lower particle density than GroEL-EG (Supplementary Fig. 4a), while those obtained from some areas of the other grid (dataset referred to as GroEL-Gh) showed densely packed and side-view oriented particles similarly to GroEL-EG (Supplementary Fig. 4b). GroEL-Gh showed more side-view preference compared to GroEL-Gl (Supplementary Fig. 4c,d), indicating that the orientation distribution of GroEL is somehow strongly affected by the particle density. To collect about 200,000 particle images of GroEL for these two datasets, we collected 5614 micrographs for GroEL-Gl and 649 for GroEL-Gh. The final reconstructions were obtained from 215,011 and 198,677 particle images, with resolutions of 2.08 and 2.09 Å for the GroEL-Gl and GroEL-Gh datasets, respectively (Fig. 2g; Supplementary Fig. 4e–f; Supplementary Table 1).

We counted the average number of particles per image contributing to the final reconstruction (NPPI-final), which would reflect the efficiency of data collection. For the GroEL-EG and GroEL-Gh datasets, 315 and 306 particles/image were used for final reconstruction, respectively, while only 36 and 38 particles/image were used for the GroEL-Q and GroEL-Gl datasets, respectively (Supplementary Table 1). Although both the GroEL-EG and GroEL-Gh datasets showed high particle density and side-preferred orientation, the resolution of the final map of GroEL-EG (1.99 Å) was slightly higher than that of GroEL-Gh (2.09 Å) (Fig. 2g; Supplementary Table 1). The number fraction of particles used for the final map in those initially picked, which we named the final/initial particle number ratio (FI ratio), should reflect the fraction of protein molecules keeping the intact structures upon blotting and quick freezing in the vitreous thin ice film. In the GroEL-EG dataset, 88.9% (158,485 out of 178,215) of the particles remained after 2D and 3D classifications, but only 50.8% (20,061 out of 39,492), 19.2% (215,011 out of 1,118,169), and 58.4% (198,677 out of 340,446) did so for the GroEL-Q, GroEL-Gl, and GroEL-Gh datasets, respectively (Supplementary Table 1). Additionally, the Rosenthal-Henderson B-factor^29^ of GroEL-EG (74.1 Å^2^) was smaller (better) than those of GroEL-Q (98.5 Å^2^), GroEL-Gl (83.3 Å^2^), and GroEL-Gh (85.4 Å^2^) (Supplementary Fig. 5a–d, left panels), demonstrating a higher homogeneity of the particle structures on the EG-grid to reach such higher resolution. We also calculated the FSC curves and sphericities of these datasets using the 3DFSC server^30^ and found that the sphericity of GroEL-EG (0.991) was slightly higher than those of GroEL-Q (0.964), GroEL-Gl (0.985) and GroEL-Gh (0.985) (Supplementary Fig. 5a–d, right panels). The efficiency (*E*_od_, calculated by cryoEF^31^), which represents the uniformity of orientation distribution used in the reconstruction (the perfect orientation distribution is presented as *E*_od_ = 1.0), of GroEL-EG (0.71) was slightly lower than those of GroEL-Gl (0.75) and GroEL-Gh (0.74) (Fig. 2c; Supplementary Fig. 4c,d) but was still high enough to achieve high-resolution reconstruction.

### GroEL particle locations within the ice layer on the EG-grid

We collected a cryo-ET dataset from the same GroEL-EG grid to investigate the locations of GroEL particles embedded in the vitreous ice film on the EG-grid. We selected an area of densely packed GroEL particles with some small ice contaminants (Supplementary Fig. 6a). It was clear from highly tilted images that these ice contaminants were located on the surface of vitreous ice (Supplementary Fig. 6b). Reconstruction of tomographic images from this dataset revealed three unique layers from the top to bottom. In the top layer, small ice contaminants were observed and no GroEL particles were found (Supplementary Fig. 6c). The middle layer 240 Å below the top layer contained densely packed GroEL particles (Supplementary Fig. 6d). Unexpectedly, at 225 Å below the middle layer, there were a few GroEL particles that seemed to be bound on the back side of the graphene layer (Supplementary Fig. 6e). The estimated distance from the air–water interface to the bottom of the major GroEL layer was ~ 160 Å, which is reasonable to accommodate a single GroEL layer (Supplementary Fig. 6f). These results indicate that GroEL particles were located close to the graphene surface and away from the air–water interface (see also Supplementary Movie 2). We also collected a cryo-ET dataset from the GroEL-Gh grid and selected a hole containing both areas with and without graphene support for comparison. The graphene area showed a high density of GroEL particles in a single layer like the EG-grid, demonstrating the compatibility with the EG-grid, while fewer particles were observed in the non-graphene area (Supplementary Fig. 7; Supplementary Movie 3).

### Storage of ClO_2_^·^ oxidized graphene grid

**Figure 3:**
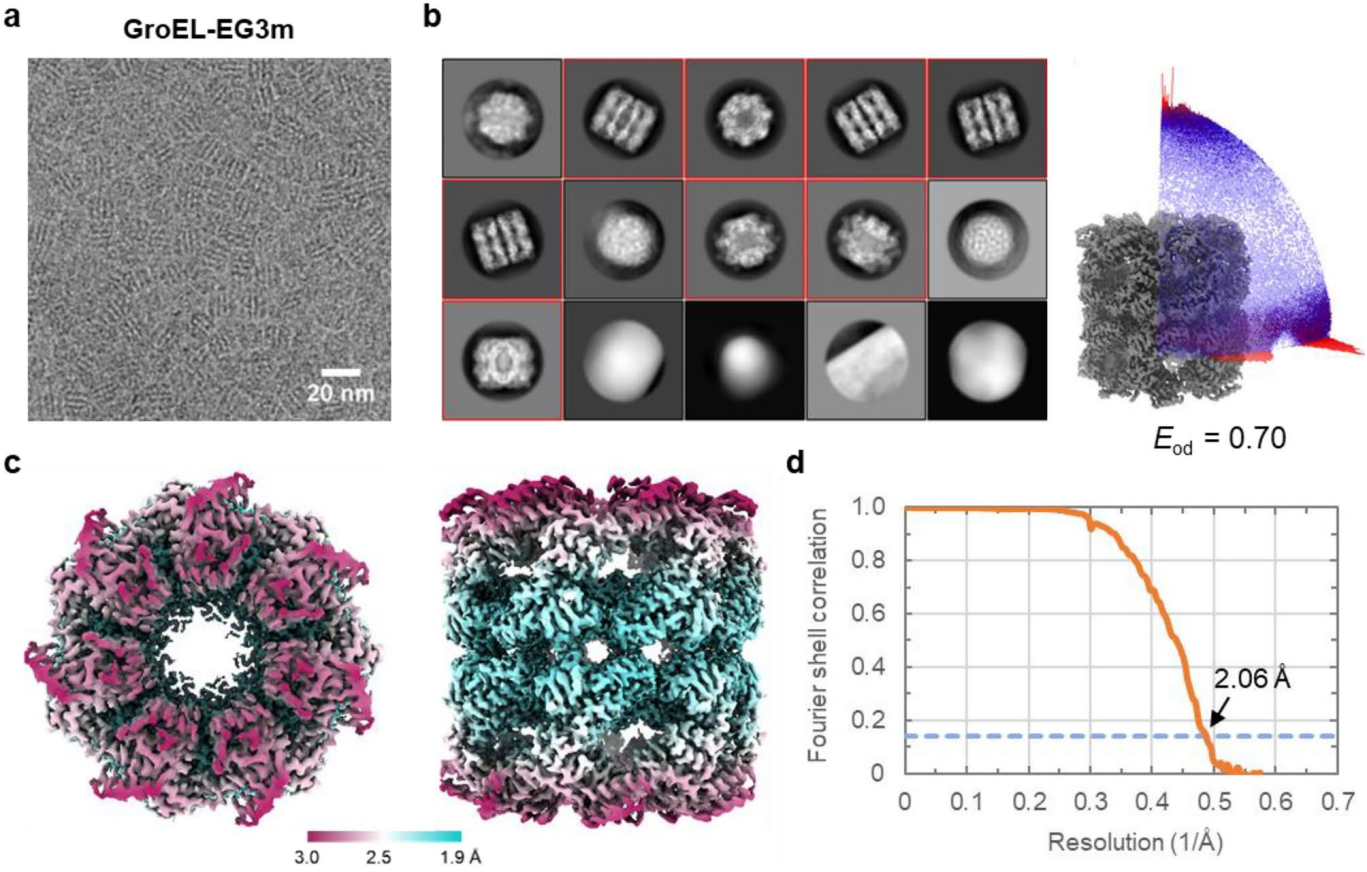
Demonstration of storability of the ClO_2_^·^ oxidized graphene grid. CryoEM image analyses of GroEL was carried out on the EG-grid produced by 5-min epichlorohydrin treatment of a ClO_2_^·^ oxidized graphene grid stored under N_2_ gas at room temperature for 3 months. (**a**) Typical cryoEM image of GroEL on the EG-grid. (**b**) Selected 2D class averages aligned in descending order of particle numbers from left to right and top to bottom. The classes selected for the following analysis are indicated with red boxes. Right panel shows the angular distribution of particles used in the final refinement. The final 3D map is shown for reference. The efficiency (*E*_od_) calculated with cryoEF is also shown. (**c**) Two orthogonal views of the final 3D map: top view, left panel; and side view, right panel. The local resolution distribution is colored as in the color bar. (**d**) The FSC curve for the final map. The dashed blue line indicates the FSC = 0.143 criterion.

We also tested whether the ClO_2_^·^ oxidized graphene grids are storable or not. After ClO_2_^·^ treatment for 10 min, the grids were washed with water, air-dried, and stored under N_2_ atmosphere at room temperature for 3 months. Then, the grids were chemically modified by 1% (v/v) ECH solution, and 3 μl of 1.5 mg ml^−1^ GroEL solution was loaded in the same way as GroEL-EG. We found many GroEL particles adsorbed on the EG-grid (Fig. 3a) and collected and processed a dataset (referred to as GroEL-EG3m). The 2D class averages and particle orientation distribution showed more side views than the other views as GroEL-EG and GroEL-Gh (Fig. 3b). The resolution of the final 3D map reconstructed from 500 micrographs reached 2.06 Å (Fig. 3c,d). The Rosenthal-Henderson B-factor was 64.9 Å^2^, which was even smaller than that of GroEL-EG albeit slightly (Supplementary Fig. 8a), and the calculated sphericity (0.976) and efficiency factor (0.70) were comparable to those of GroEL-EG (Fig. 3b; Supplementary Fig. 8b), ensuring the long storability of the ClO_2_^·^ oxidized graphene grids. The lower FI ratio (33.6%) and NPPI-final (104.1) compared to the GroEL-EG dataset (Supplementary Table 1) may reflect the way of the data collection, because, unlike the GroEL dataset described above, we collected three micrographs from one hole in the GroEL-EG3m, leading to carbon areas included in some micrographs to cause many particles on the carbon areas picked automatically.

### CryoEM image analysis of SARS-CoV-2 spike protein on the EG-grid

**Figure 4:**
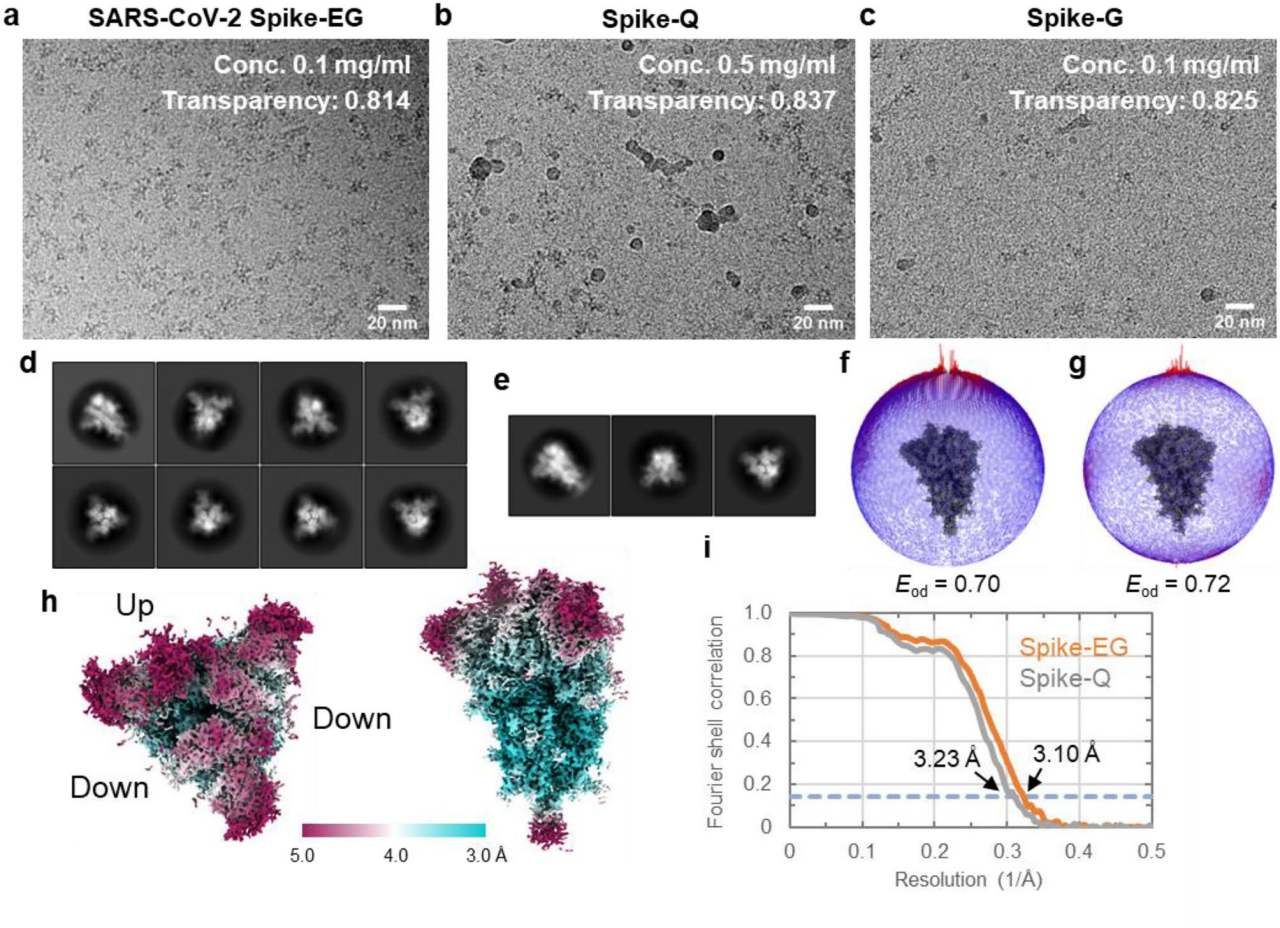
CryoEM image analyses of SARS-CoV-2 spike protein on the EG-grid and Quantifoil grid. (**a**–**c**) Typical cryoEM images of spike protein on the EG-grid (spike-EG) (**a**), on the Quantifoil grid (spike-Q) (**b**), and on the glow discharged graphene grid (spike-G) (**c**). The protein concentration and the value of electron beam transparency of each image (measured from the image brightness ratio between the energy filter slit in and out) are also shown. (**d** and **e**) Selected 2D class averages from the spike-EG dataset (**d**) and the spike-Q dataset (**e**). The class averages are aligned in descending order of particle numbers from left to right and top to bottom. (**f** and **g**) Angular distributions of particles used in the final refinement for the spike-EG dataset (**f**) and the spike-Q dataset (**g**). Final 3D maps are shown for reference. The efficiencies (*E*_od_) calculated with cryoEF are also shown. (**h**) Two orthogonal views of the final 3D map from the spike-EG dataset: top view of the trimer, left panel; and side view, right panel. The local resolution distribution is colored as in the color bar. (**i**) The FSC curves for the final maps of the spike-EG and spike-Q dataset. The dashed blue line indicates the FSC = 0.143 criterion.

Next, we examined the performance of the EG-grid on the SARS-CoV-2 spike protein trimer (*M*_w_ ~ 420 kDa), for which the present demand for high-resolution structures is quite high. We prepared three specimen grids: EG-grid (referred to as Spike-EG) and glow discharged graphene grid (referred to as Spike-G), both at a protein concentration of 0.1 mg ml^−1^, and conventional Quantifoil grid (referred to as Spike-Q) at a protein concentration of 0.5 mg ml^−1^. None of the grids were washed with the buffer after protein loading. The particle density was much higher in Spike-EG than that in Spike-Q, although the protein concentration was five times lower for Spike-EG (Fig. 4a,b). The particle density in Spike-G was too low to collect a dataset even in the area of ice thicknesses similar to those of Spike-EG and Spike-Q (Fig. 4c). The level of image variety of the 2D class averages, the uniformity in the orientation distribution and calculated efficiency (*E*_od_) were all similar in Spike-EG and Spike-Q (Fig. 4d–g). The 3D maps reconstructed from these two datasets both showed a conformation of the receptor binding domain (RBD) with 1-up and 2-down (Fig. 4h; Supplementary Fig. 9a). Particle images of 150,316 and 58,102 used for the final reconstructions were picked up from 1163 and 1029 micrographs, resulting in resolutions of 3.10 Å and 3.23 Å for the Spike-EG and Spike-Q dataset, respectively (Fig. 4i; Supplementary Table 1). The Rosenthal-Henderson B-factors of these datasets were also similar to each other (107.4 Å^2^ for Spike-EG and 101.9 Å^2^ for Spike-Q) (Supplementary Fig. 9b,c). The value of NPPI-final for Spike-EG (129.2) was about twice as much as that for Spike-Q (56.5), while the FI ratio values were quite similar (24.9% and 23.8%) (Supplementary Table 1).

### CryoEM analysis of V_1_-ATPase, GAPDH, β-galactosidase, and apoferritin on the EG-grid

**Figure 5:**
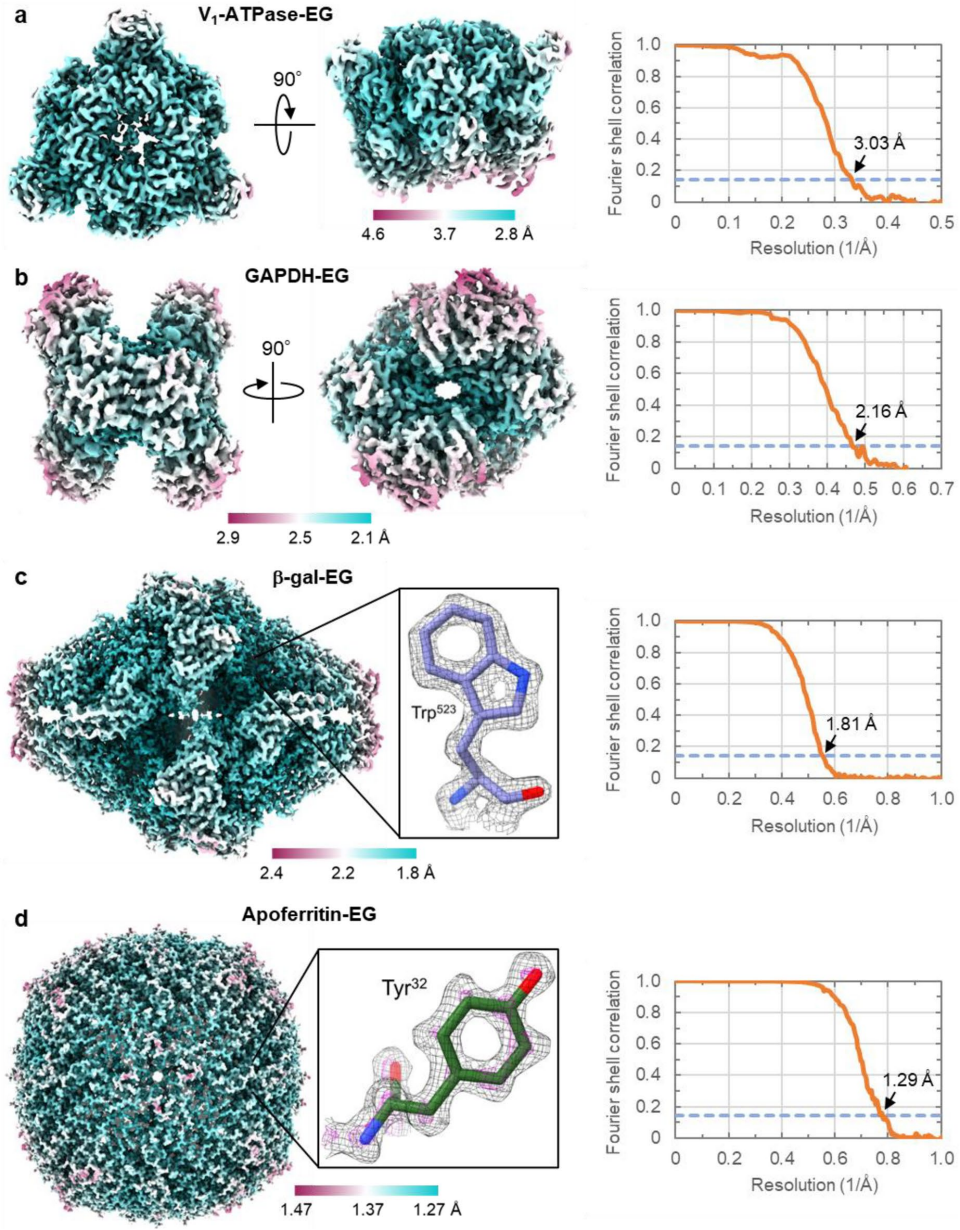
CryoEM analyses of the A_3_B_3_ ring of V_1_-ATPase, GAPDH, β-galactosidase, and apoferritin on EG-grids. (**a**–**d**) The final 3D maps from the V_1_-ATPase-EG (**a**), GAPDH-EG (**b**), β-galactosidase-EG (**c**), and apoferritin-EG (**d**) dataset. Two orthogonal views are shown in (**a**) and (**b**). The insets show the density maps and the fitted models of β-galactosidase (PDB entry: 6X1Q) around Trp^523^ (**c**) and apoferritin (PDB entry: 6V21) around Tyr^32^ (**d**). The map in (**d**) is shown in two contour levels of 0.05 and 0.15 in black and magenta, respectively. The local resolution distributions are colored as in the color bars. Right panels show the FSC curves for the final maps. The dashed blue lines indicate the FSC = 0.143 criterion.

We applied the EG-grid to four samples: the chimeric complex of the soluble domain of V/A-type ATPase (V_1_-ATPase) composed of the A_3_B_3_ hexametric ring from *Thermus thermophilus* and the DF subunits from *Homo sapiens* (*M*_w_ ~ 400 kDa), the tetramer of glyceraldehyde 3-phosphate dehydrogenase (GAPDH) (*M*_w_ ~ 144 kDa), β-galactosidase (β-gal) (*M*_w_ ~ 460 kDa), and apoferritin (*M*_w_ ~ 500 kDa). The GAPDH structure has not been solved by cryoEM except for the part of the large ternary complex^32^. We loaded each sample solution onto an EG-grid and made a cryoEM grid by plunge freezing in the same way as we did for GroEL. In all samples, we saw many protein particles in the micrographs (Supplementary Figs. 10a, 11a, 12a, and 13a). Datasets were collected and processed (referred to as V_1_-ATPase-EG, GAPDH-EG, β-gal-EG, and apoferritin-EG). The 2D class averages showed wide varieties in particle orientations (Supplementary Figs. 10b, 11b, 12b, and 13b). During 3D classification of V_1_-ATPase-EG, we found two classes, one with and the other without the human DF subunits corresponding to the central rotary shaft. We only selected the class without the shaft, because the particle number of this class was much larger than that of the other. The 3D map and model with the shaft will be presented elsewhere. The orientation distributions of the particles used for the final maps were quite uniform as expected from the 2D class averages (Supplementary Figs. 10c, 11c, 12c, and 13c). The overall map resolutions of V_1_-ATPase, GAPDH, β-galactosidase, and apoferritin reached 3.03 Å, 2.16 Å, 1.81 Å, and 1.29 Å. respectively (Fig. 5a–d). We also tried to reconstruct a 3D map of GAPDH on a Quantifoil grid (referred to as GAPDH-Q) but failed due to a severe preferred orientation derived from particle segregation (Supplementary Fig. 11d,e). The central region of the β-gal-EG density map had a sufficiently high resolution and quality to show the holes in the center of two aromatic rings of Trp residues (Fig. 5c, inset). The density map of apoferritin-EG also had a sufficiently high resolution and quality to display individual atoms as blobs at a relatively high contour level, as indicated by the contour in magenta for a Tyr residue (Fig. 5d, inset). The overall resolution of β-gal-EG is almost the same as the highest resolution 1.82 Å of the map deposited in the EMDB to date, which was reconstructed from 257,202 particles extracted from 4949 images^33^. The Rosenthal-Henderson B-factors of β-gal-EG (56.6 Å^2^) and apoferritin-EG (36.4 Å^2^) are also as small as those of the previous reconstructions^34,35^ (Supplementary Figs. 12d and 13d). So the EG-grid allowed to reach the highest resolution of β-galactosidase with 40% less number of micrographs. The FI ratios and the NPPI-finals of V_1_-ATPase, GAPDH, β-galactosidase, and apoferritin were 20.0%, 29.4%, 47.0%, and 66.5%, and 116.4, 368.2, 71.4, and 70.3, respectively (Supplementary Table 1).

We also applied the EG-grid to hemagglutinin (HA), which suffered from preferred orientation and needed grid tilting data collection for 3D reconstruction^30^. HA on the EG-grid showed a large number of particles on each micrograph (Supplementary Fig. 14a) and 2D class averages with an improved orientation distribution (many side views, Supplementary Fig. 14b), although the resolution of the 3D map was limited to 6–7 Å.

## Discussion

Oxidation of graphene films on EM grids without damaging is not an easy task. In fact, the plasma method (glow discharge) has been reported to cause graphene to collapse in a short time^19^. It has also been reported that UV/O_3_ or KMnO_4_ treatment leads to the introduction of carboxy groups, implying the cleavage of C=C bonds in graphene, and that was confirmed by the formation of nanopores^13^. As can be seen in these reports, it is difficult to oxidize chemically stable graphene to introduce oxygen functional groups on the surface without disruption of its atomic layer sheet structure. The distinctly different results of GroEL-Gl and GroEL-Gh obtained by plasma-treated graphene grids (Supplementary Fig. 4a–d) also indicate the difficulty in controlling the property of graphene surface by plasma treatment to reproduce good results in the cryoEM grid preparation.

We have successfully developed a novel oxidative modification method of graphene on substrates using photoactivated ClO_2_^·^. This method can introduce reactive functional groups such as hydroxy (OH) group, while maintaining the graphene framework intact. In our method, C–O single bond groups (hydroxy groups) are more preferentially introduced compared to C=O type functional groups such as carboxy groups (Supplementary Fig. 1a–b), and therefore the intrinsic sheet structure of graphene is better maintained. We also confirmed that these hydroxyl groups react with ECH to produce epoxy groups and that they further react with amine compounds (Supplementary Fig. 1c). CryoEM observation confirmed that little damage was introduced to the graphene after oxidation, epoxidation, and vitrification (Supplementary Fig. 2f.). This suggests not only a variety of applications of the EG-grid but also a much wider potential application of the method we used for graphene oxidation by combination with further chemical modifications.

We also confirmed that ClO_2_^·^ oxidized graphene grids can be stored for at least 3 months under N_2_ atmosphere at room temperature (Fig. 3a–d), which enables stable supply of the oxidized graphene grids in a package to users so that users need to perform only the 5-min epoxidation reaction before use. We are now exploring storage conditions of the oxidized graphene grid and EG-grid for longer storage by the combination of different storage conditions, such as gaseous environment and temperature. As one remaining challenging step for mass production is the preparation of high-quality, ultraclean CVD graphene grids without contaminations such as residual PMMA, the fabrication method recently developed by Zheng et al.^36^ would be of great help.

As we tested the EG-grid for many samples, we found that the effectiveness of the EG-grid somewhat varied from sample to sample. For GroEL, both GroEL-EG and GroEL-Gh showed densely packed particles and large values for NPPI-final (Fig. 2a; Supplementary Fig. 4b and Supplementary Table 1), which means that many particles were kept attached even after buffer washing in some areas of the graphene surface even without epoxidation. The high-density adsorption of GroEL particles on the graphene surface may make it resistant to buffer washing. In addition, the orientation distribution of GroEL on a cryoEM grid was strongly affected by the particle density rather than the epoxidation of graphene. However, the biggest difference between the EG-grid and hydrophilized graphene grid was the values of FI ratio (88.9% for GroEL-EG and 58.4% for GroEL-Gh). We suspect that the much higher FI ratio of GroEL-EG might reflect the intactness of protein particles possibly by particles being kept slightly away from the graphene surface with the short linker introduced by epoxidation, which in turn results in the better Rosenthal-Henderson B-factor and better resolution of the final 3D map (1.99 Å). Furthermore, the epoxy-introduced surface of the EG-grid may offer some extent of hydrophobicity (Fig. 1a), and the surface property with a well-balanced combination of hydrophilicity and hydrophobicity realized on the EG-grid may be important for “gentle” adsorption of protein particles on the surface, as demonstrated by the much higher value of FI ratio in the GroEL-EG dataset than the GroEL-Gl and GroEL-Gh datasets (Supplementary Table 1).

Another example demonstrating the effectiveness of the EG-grid is SARS-CoV-2 spike protein. The Spike-EG images showed much higher particle density at a very low concentration (0.1 mg ml^−1^) compared to those of Spike-Q and Spike-G (Fig. 4a–c). Although the overall resolution of the final 3D map was not dramatically improved by using the EG-grid, the EG-grid was still quite beneficial in terms of saving the amount of sample and the time for grid screening and data collection, especially for those requiring many structural analyses of the complexes of a target protein with different compounds and/or antibodies^37^. In other examples, GAPDH-EG represented markedly improved orientation distribution of the particles (Supplementary Fig. 11a–d) and also proved that near 2 Å resolution is reachable with ~ 144 kDa protein only from ~ 240 images (Fig. 5b; Supplementary Table 1), which can now be collected in about half an hour. This result indicates that the biased orientation to the side view in GroEL-EG is a special case due to the cylindrical shape of the GroEL particle, but generally speaking, the orientation distribution of globular proteins tends to be more uniform, such as that shown in the case of GAPDH where it was dramatically improved with the EG-grid. In case the particle orientation distribution on the EG-grid is distinct from that on the Quantifoil grid, merging the two datasets will help to reach higher resolution. The β-gal-EG and apoferritin-EG datasets both demonstrated the capability of the EG-grid for high resolution structure determination beyond 2 Å without any problem (Fig. 5b,c), and that the resolution of apoferritin easily reached 1.29 Å resolution indicates its atomic-resolution imaging capability (Fig. 5d). Of course, there may be some cases where the EG-grid is still not effective enough to reach high resolution such as HA (Supplementary Fig. 14a–b). One major concern is the residual PMMA contamination on the graphene surface that increases the background, and this may be critical for high-resolution cryoEM imaging of small proteins. Another possibility is that hemagglutinin particles were partially denatured on the surface of the EG-grid, especially in the side views.

In summary, the parameters indicating the superiority of the EG-grid seem to vary from protein to protein, but the clear and general advantage of the EG-grid is that much more protein particles can be observed in the same size of viewing area with a more uniform orientation distribution than conventionally used holey carbon grids, and in most cases, one-time grid preparation is sufficient, greatly reducing the time required for grid screening for cryoEM data collection for high-resolution 3D reconstruction, especially for the samples suffering from low yield, poor particle density, and preferred orientation. Moreover, the ClO_2_^·^ oxidized graphene surface can be chemically modified easily in many different ways to develop different types of grids with different surface functionalities, which would further expand the potential of cryoEM towards much wider applications, for example, by allowing easier and quicker on-the-grid sample purification. Such developments are under way.

## Methods

### Graphene grid fabrication

We prepared graphene grids using Quantifoil grids based on the protocol described previously with some modifications^13^. PMMA-coated CVD graphene, either 2, 4 or 6-layers, on a Cu foil (AirMembrane), or PMMA-coated single-layer CVD graphene on a Cu foil from any suppliers (ACS Material, Graphenea, SIGMA-Aldrich etc.) can be used. If necessary, the backside (the opposite side of PMMA) of graphene was removed by mechanical polishing using a waterproof abrasive paper (P3000, NIHON KENSHI Co., Ltd.). The Cu foil was etched by floating the PMMA-coated CVD graphene on a 0.5 M ammonium persulfate (APS) solution for more than 30 min. Then, the PMMA-coated graphene was scooped up on a filter paper, transferred to water and left for 10 min. This washing process was repeated twice. Or, a much easier and quicker method is to use Trivial Transfer Graphene™ (ACS Material), which can be transferred to water directly without polishing, etching, and washing. The graphene was transferred to water in a stainless container in which a number of Quantifoil grids (R1.2/1.3 Au 200 mesh) were aligned at the bottom with their carbon side facing up. The water was slowly drained, and the graphene layer was deposited on the grids. After air-dried for more than 1 h, each grid was separated and baked at 130 °C for 15 min. The grids were immersed in acetic acid twice for PMMA removal: for 2–3 h in the first step and then overnight. Then the grids were soaked in isopropanol for 10 min, air-dried on a filter paper, and baked at 100 °C for 10 min. The grids were stored in a grid box in a desiccator.

### Oxidation of graphene grid

The graphene grids prepared as above were oxidized by photoactivated ClO_2_^·^ gas generated from an aqueous solution (20 ml) of NaClO_2_ (200 mg) and 37% HCl (aq) (100 μl) by irradiation with an UV LED lamp (λ = 365 nm, 20 mW/cm^2^) for 10 min. A square-shaped dish-like glassware with an inner and outer compartment that we developed before (shown in Fig. 6 of^38^) was used to perform this oxidation process. The aqueous solution of NaClO_2_ was filled into the outer compartment, the graphene grids were laid in the inner compartment, and a glass plate was put on the glassware to seal the entire chamber. To avoid any damage on the graphene layer by direct photoirradiation, an aluminum foil was laid on the grids to shield them from the UV light (see also Supplementary Movie 1). The total reaction volume can be reduced to 5 ml. For the storage experiment (GroEL-EG3m), the ClO_2_^·^ oxidized graphene grids were washed with 5 μl of ultrapure water three times, air-dried, and stored under N_2_ gas at room temperature for 3 months. The graphene on a silicon wafer were also oxidized in the same way. Because the ClO_2_^·^ gas is toxic and potentially explosive especially at high concentration, all the oxidation process should be performed under controlled concentration in a fume hood. Plasma treated hydrophilized graphene on a silicon wafer were also prepared as a control for XPS measurement by glow discharge (30 mA, 20 s) using a JEC-3000FC sputter coater (JEOL) with an aluminum foil mask to shield them from plasma irradiation.

### Fluorination of graphene grid

The preparation of EG-grid (surface functionalization of oxidized graphene by 1% ECH) was performed as described in manuscript for negative staining. A 3 μl aqueous solution of 1% 1H,1H-undecafluorohexylamine (UFHA) was loaded on the EG-grid. After 5 min, the solution was blotted with a filter paper and the grid was washed with 5 μl water for 3 times. After washing process, the fluorinated graphene grid was dried in vacuo overnight.

### Surface analysis of grapheme

Raman spectra were obtained with an NRS-3100 T laser Raman spectrometer (JASCO) with a 532 nm laser at room temperature. Fourier-transform infrared (FTIR) spectroscopic measurements were performed using a Spectrum Two spectrometer (Perkin-Elmer) equipped with an UATR two attachment. All the spectra were acquired at a resolution of 4 cm^−1^ over 16 scans in a scan range of 600–4000 cm^−1^. The surface chemical composition of the graphene was determined using a JPS-9010MC XPS instrument (JEOL). The XPS insert parameters included the power of analysis (wide: 75 W, narrow: 150 W) and monochromatic Mg K_α_ radiation. The survey and high-resolution XPS profiles were obtained at the constant analyzer pass energies of 160 and 10 eV, respectively. The CasaXPS Version 2.3.15 software was used for the peak-differentiation-imitating analysis of the C1s narrow XPS profile. Two independent experiments were conducted to obtain the standard deviation of the data. The static water contact angles of the graphene surfaces were determined using a Drop Master DM300 (Kyowa Interface Science) contact angle meter. A 1.0 μl of water droplet was placed on the surface of the graphene on the grid, and the contact angle was determined 5 s (scanning time) after the attachment of the droplet. Data are obtained as the mean of three independent experiments with standard deviation. All the cryoEM images and diffraction patterns of the grids were acquired using SerialEM^39^ and JEM-Z300FSC (CRYO ARM™ 300, JEOL) operated at 300 kV with a K3 direct electron detector (Gatan, Inc.). The peak values of the diffraction intensities were identified using Fiji^40^.

### Protein preparation

GroEL from *Escherichia coli* was purchased from TaKaRa Bio Inc. (Product code 7330). A 5 mg powder sample of GroEL was dissolved with 500 μl of GroEL buffer (25 mM HEPES pH 8.0, 50 mM NaCl), and it was purified using a Superdex 200 Increase 10/300 GL gel filtration column with the GroEL buffer. The main peak was collected and concentrated to 3.2 mg ml^−1^, flash-frozen with liquid nitrogen and stored at − 80 °C.

The codon-optimized gene of SARS-CoV-2 Spike (S) protein (GenBank: QHD43416) was designed for expression in mammalian cells and synthesized from GeneArt DNA Synthesis (Thermo). For expression of recombinant S protein, the sequence encoding the S ectodomain (residues 1–1208) with proline substitutions at residues 986 and 987, a “GSAS” substitution at furin cleavage site (residues 682–685), and C-terminal foldon trimerization motif followed by an octa-histidine tag was cloned into a pcDNA3.1 expression vector (Invitrogen). Further, S protein D614G mutation was introduced by inverse PCR method. Recombinant S proteins were transiently expressed in Expi293f. cells (Thermo) maintained in HE400AZ medium (Gmep, Japan). The expression vector was transfected by using Gxpress 293 Transfection Kit (Gmep, Japan) as following the manufacturer’s protocol. After 5 days post-transfection, culture supernatants were harvested, and His-tagged S proteins were purified by Ni^2+^ affinity chromatography using Ni Sepharose 6 Fast Flow (Cytiva), followed by size exclusion chromatography using Superdex 200 Increase 10/300 GL (Cytiva) equilibrated with a buffer containing 50 mM HEPES (pH 7.0) and 200 mM NaCl.

*Escherichia coli* strain BL21-CodonPlus-RP (Stratagene) was used for expression of the human DF-V_1_-ATPase chimeric complex. The recombinant complex was isolated as described previously^41,42^. The expression plasmids for V_1_-ATPase containing DF from *Homo sapiens* were constructed by the same method as described previously^41^. The genes encoding the D and F subunits were amplified from human cDNA. The amplified fragment was then digested with appropriate restriction enzymes and inserted into the corresponding region of the *Thermus thermophilus* V_1_-ATPase expression plasmid^42^. The mutant V_1_-ATPase (A-His8/ΔCys, A-C255A/A-S232A/A-T235S, FS54C) was used for preparation of the complex.

The gene of human GAPDH was cloned into pET-28a(+) expression vector (Novagen) with an N-terminal His-tag followed by the Tobacco Etch Virus (TEV) protease cleavage site. The recombinant protein was expressed using an *Escherichia coli* strain, BL21 Star™ (DE3) (Thermo Fisher Scientific). The transformed cells were cultured in Luria–Bertani (LB) medium with 50 μg ml^−1^ Kanamycin at 37 °C until the OD_600_ reached ~ 0.6. After adding 1 mM isopropyl-β-D-thiogalactopyranoside (IPTG) and culturing for 16 h at 37 °C, cells were harvested by centrifugation, flash-frozen with liquid nitrogen, and stored at − 80 °C. Frozen bacteria pellets were resuspended in a buffer containing 20 mM Tris–HCl pH 8.0, 30 mM NaCl, 1 mM 1,4-dithiothreitol (DTT) supplemented with 10 μl Benzonase, 1 mg ml^−1^ lysozyme, and EDTA-free cOmplete™ protease inhibitor cocktail (Roche) and lysed by sonication. The suspension was centrifuged for 30 min at 200,000×*g* and 4 °C. The supernatant was collected and purified using a HisTrap HP 5 ml column (GE Healthcare) equilibrated with a buffer containing 20 mM Na_4_P_2_O_7_ pH 7.4, 500 mM NaCl, 30 mM imidazole, and subsequently eluted with the same buffer containing 500 mM imidazole. The desired fractions were collected and dialyzed overnight at 4 °C against a buffer composed of 20 mM Na_4_P_2_O_7_ pH 7.4, 200 mM NaCl supplemented with TEV protease. The protein was further purified using a HiLoad 16/600 Superdex 200 column (GE Healthcare) equilibrated with 20 mM Tris–HCl pH 8.0, 30 mM NaCl, 1 mM Tris(2-carboxyethyl)phosphine (TCEP). The desired fractions were collected, concentrated to ~ 10 mg ml^−1^, flash-frozen with liquid nitrogen, and stored at − 80 °C.

β-galactosidase (β-gal) was purchased from SIGMA-Aldrich (Product code G5635) and dissolved with β-gal buffer (25 mM HEPES pH 8.0, 50 mM NaCl, 2 mM MgCl_2_, 1 mM TCEP) to a final concentration of 4.0 mg ml^−1^. IPTG was supplemented to a final concentration of 5 mM, and the solution was incubated on ice for 30 min. β-gal was purified using Superdex 200 Increase 10/300 GL column (GE Healthcare) with the same buffer. The main peak was collected and concentrated to 2.0 mg ml^−1^, flash-frozen with liquid nitrogen and stored at − 80 °C.

Mouse apoferritin was expressed using mFth1-pET24a plasmid and an *Escherichia coli* strain, BL21 Star™ (DE3) (Thermo Fisher Scientific). After the plasmid was transformed, the cells were cultured in 4 ml of LB medium with 50 μg ml^−1^ Kanamycin and 0.5% glucose overnight. The culture was transferred into 500 ml of fresh culture medium and cultured at 37 °C until the optical density at 600 nm reached 0.5. Then, 1 mM IPTG was added, and the cultivation was continued for 4 h at 37 °C. Cells were harvested and resuspended in buffer A (30 mM HEPES pH 7.5, 300 mM NaCl, 1 mM MgSO_4_) supplemented with 1 mg ml^−1^ lysozyme and lysed by sonication. The suspension was centrifuged, and the supernatant was incubated at 70 °C for 10 min. After centrifugation and collecting supernatant again, the protein was precipitated by adding ammonium sulfate to a final concentration of ~ 53% and stirring on ice for 30 min. After centrifugation and collecting pellet, the pellet was washed with buffer A supplemented with ~ 53% ammonium sulfate. This washing step was repeated twice. The washed pellet was resuspended in 2 ml of phosphate buffered saline (PBS) and dialyzed against 500 ml of PBS at 4 °C overnight. Precipitation was removed by ultracentrifugation, and the solution was concentrated to ~ 0.9 ml, which was further purified using a Superose 6 10/300 column (GE Healthcare) equilibrated with 20 mM HEPES pH 7.5, 150 mM NaCl. The desired fractions were collected separately, flash-frozen with liquid nitrogen and stored at − 80 °C.

Influenza hemagglutinin trimer (HA) (H3N2, A/Hong Kong/1/1968) was purchased from MyBioSource (catalog number: MBS434205) and dissolved in 1× PBS buffer to a concentration of 0.5 mg ml^−1^.

### Epoxidation of oxidized graphene grid and negative staining

A 3 μl solution of 1% (v/v) epichlorohydrin (ECH) in water was loaded on the graphene side of an oxidized graphene grid held with a tweezer. After 5 min, the ECH solution was blotted with a filter paper, and the grid was washed with 5 μl of ultrapure water (or protein buffer not containing a compound with amino group such as Tris) three times. Then, 3 μl of GroEL solution (0.3 mg ml^−1^) was loaded on the graphene side of the grid. After 5 min, the protein solution was blotted, and the grid was washed with 5 μl of the same buffer three times (see also Supplementary Movie 1). Then the grid was immediately stained with 3 μl of 2% uranyl acetate solution. Images were taken using a JEM-1011 electron microscope (JEOL) at 100 kV.

### CryoEM grid preparation

The surface functionalization of oxidized graphene by 1% ECH and the protein loading step were performed as described above for negative staining. Protein concentration was 1.5 mg ml^−1^ for GroEL-EG, GroEL-EG3m, and the β-gal-EG, 0.1 mg ml^−1^ for the SARS-CoV-2 Spike-EG, 1.0 mg ml^−1^ for the V_1_-ATPase-EG, 0.5 mg ml^−1^ for GAPDH-EG and HA, and 1.8 mg ml^−1^ for apoferritin-EG. For SARS-CoV-2 spike protein, the stock sample was diluted with the buffer containing 20 mM HEPES (pH 7.4) and 150 mM NaCl. In the washing step after protein loading, the final (third) washing buffer on the grid was not blotted, and the tweezer holding the grid was placed in a chamber of Vitrobot™ Mark IV (Thermo Fisher Scientific) equilibrated at 4 °C and 100% humidity. For Spike-EG, GAPDH-EG, and HA, the grid was not washed after the protein loading. After a liquid ethane container was placed, the grid was blotted for 1.5 s (for all samples other than Spike-EG) or 2.0 s (for Spike-EG) and immediately plunged into liquid ethane. Excessive ethane was manually blotted with a filter paper, and the grid was stored in liquid nitrogen. For comparison, non-oxidized graphene grid (for GroEL-Gl, GroEL-Gh, and Spike-G) or Quantifoil grids (R1.2/1.3 Cu 200 mesh, for GroEL-Q, Spike-Q, and GAPDH-Q) was hydrophilized by glow discharge using a JEC-3000FC sputter coater (JEOL). To avoid graphene breakage, a loose mask made of a thin aluminum foil was placed above the grids during the glow discharge for the graphene grid. A tweezer holding the grid was placed in a chamber of Vitrobot™ and after the liquid ethane container was placed, 3 μl of 1.5 mg ml^−1^ (for GroEL-Q and GroEL-Gl) or 1.0 mg ml^−1^ (for GroEL-Gh) GroEL, or 0.1 mg ml^−1^ (for Spike-G) or 0.5 mg ml^−1^ (for Spike-Q) spike protein, or 0.5 mg ml^−1^ (for GAPDH-Q) GAPDH solution was loaded on the graphene or carbon side. Then the grid was blotted for 1.5 s (for GroEL-Gl and GroEL-Gh) or 2.0 s (for GroEL-Q, Spike-G, and Spike-Q) or 3.0 s (for GAPDH-Q) and immediately plunged into liquid ethane. Excessive ethane was manually blotted with a filter paper, and the grid was stored in liquid nitrogen.

### CryoEM data collection for SPA

All the cryoEM image datasets were acquired using SerialEM^39^, yoneoLocr^43^, and JEM-Z300FSC (CRYO ARM™ 300, JEOL) operated at 300 kV with a K3 direct electron detector (Gatan, Inc.) in the CDS mode. The Ω-type in-column energy filter was operated with a slit width of 20 eV for zero-loss imaging. For the GroEL-EG, GroEL-Gl, GroEL-Gh, GroEL-EG3m, and GAPDH-EG dataset, nominal magnification was 60,000×. Defocus varied between − 0.5 and − 2.0 μm. Each movie was fractionated to 40 frames (0.07 s each, total exposure 2.8 s) with a total dose of 40 e^−^/Å^2^ for the GroEL-EG, GroEL-Gl, GroEL-Gh, and GAPDH-EG dataset. For the GroEL-Q, GroEL-EG3m, and GAPDH-Q dataset, three movies were collected from one hole, and each movie was fractionated to 40 frames (0.083 s each, total exposure 3.3 s for GroEL-Q, 0.063 s each, total exposure 2.6 s for GroEL-EG3m, and 0.081 s each, total exposure 3.2 s for GAPDH-Q) with a total dose of 40 e^−^/Å^2^. For the Spike-EG, Spike-Q, and HA dataset, nominal magnification was 60,000×. Defocus varied between − 0.5 and − 2.0 μm. Each movie was fractionated to 60 frames (0.05 s each, total exposure 3.0 s) with a total dose of 60 e^−^/Å^2^. For the V_1_-ATPase-EG dataset, nominal magnification was 50,000×. Defocus varied between − 1.0 and − 2.5 μm. Each movie was fractionated to 48 frames (0.084 s each, total exposure 4.0 s) with a total dose of 62 e^−^/Å^2^. For the β-gal-EG dataset, nominal magnification was 100,000×. Defocus varied between − 0.5 and − 2.0 μm. Each movie was fractionated to 40 frames (0.065 s each, total exposure 2.6 s) with a total dose of 40 e^−^/Å^2^. For the apoferritin-EG dataset, nominal magnification was 120,000×. Defocus varied between − 0.3 and − 1.3 μm. Each movie was fractionated to 64 frames (0.025 s each, total exposure 1.6 s) with a total dose of 40 e^−^/Å^2^. More parameters are summarized in Supplementary Table 1.

### SPA image processing

The movies were motion corrected using MotionCor2^44^, and CTFs were estimated using CTFFIND 4.1^45^. All the following steps were performed using RELION 3.1^46^ except GroEL-Q, GroEL-EG3m, and GAPDH-Q. RELION 4.0^47^ was used for processing for GroEL-Q, GroEL-EG3m, and GAPDH-Q. Only the images whose CTF max resolutions were beyond 5 Å were selected, and the resulting number of images are referred to as Initial images. For GroEL-EG3m, 1209 movies were processed, and 500 micrographs were randomly selected after the CTF max resolutions beyond 5 Å selection and processed in the following steps. Laplacian of Gaussian (LoG)-based auto-picking was performed for small number of images to generate the 2D class averages, which were then used as a template for second auto-picking for all images. Picked particles were extracted with 1–4× binning, and the resulting number of particles was referred to as the initial particle number. Several rounds of 2D classification were carried out to remove bad particles. For GroEL-EG3m, one round of 2D classification was performed. Initial 3D model was generated with C1 symmetry, and then D7 (for GroEL) or D2 (for GAPDH and β-gal) symmetry was applied to the resulting maps. No symmetry was applied for SARS-CoV-2 spike protein and V_1_-ATPase. 3D classification (into 4 classes) with C1 symmetry was conducted to exclude bad particles, and the number of remaining particles was referred to as the final particle number except the GAPDH-EG and β-gal-EG datasets. The remaining particles were re-extracted with a larger box size, and 3D auto-refine applying D7 (for GroEL) or D2 (for β-gal) symmetry was performed. After a soft mask around the map was generated, the resulting map was post-processed and sharpened. After several rounds of CTF refinement and Bayesian polishing with divided optics groups of 6 (for GroEL-Q), 8 (for GAPDH-EG), 10 (for GroEL-EG, GroEL-EG3m, and Spike-Q), 11 (for GroEL-Gl and V_1_-ATPase-EG), 12 (for Spike-EG), 13 (for GroEL-Gh), and 16 (for β-gal-EG), 3D auto-refine and post-processing were conducted again. For the GAPDH-EG and β-gal-EG datasets, particles were re-selected with rlnMaxValueProbDistribution = 0.05 criteria (the number of remaining particles was referred to as the final particle number), and 3D auto-refine and post-processing were conducted to generate the final map.

For apoferritin, in total 793,398 particles picked by using 2D class averages template were extracted from 7500 images (Supplementary Table 1) with a box of 512 × 512 pixels and divided into 25 groups as optical groups according to the time of data collection. After the particle selection by 2D classification, an initial model was generated, and the selected particles were subjected to several rounds of 3D auto-refine with O symmetry and post-processing with CTF refinement and Bayesian polishing. Then the particles were regrouped into 12 by the range of beam tilt X, Y (0.1 milli-radian increments) and further regrouped into 300 (= 12 × 25:5 × 5 multi-hole shots) by image shift. Regrouped particles were subjected to another round of CTF refinement, 3D auto-refine, and post-processing. Then the particles were further selected with rlnMaxValueProbDistribution = 0.04 criteria, and the selected 527,261 particles were subjected to another rounds of Bayesian polishing with a larger box size (600 × 600), 3D auto-refine, and post-processing. Finally, the Ewald sphere correction and another round of post-processing was applied to generate the final map.

The map resolutions were estimated by the gold-standard Fourier shell correlation (FSC = 0.143) criterion. The FSC curves and sphericities were calculated using 3DFSC server^30^ (https://3dfsc.salk.edu). The efficiencies in particle orientational distributions were calculated using cryoEF^31^. The Rosenthal-Henderson B-factors^29^ were calculated and plotted using bfactor_plot.py script in RELION^34^. Structural analysis and figure preparation were performed using UCSF Chimera^48^ and ChimeraX^49^.

### Tomography

The datasets for tomography were acquired for the same grid as the GroEL-EG or GroEL-Gh dataset was collected using JEM-Z300FSC (CRYO ARM™ 300, JEOL) operated at 300 kV with a K3 direct electron detector (Gatan, Inc.) in CDS mode. The Ω-type in-column energy filter was operated with a slit width of 20 eV for zero-loss imaging. For GroEL-EG, the nominal magnification was 40,000×, corresponding to 1.25 Å/pixel. Defocus was set to − 3.0 μm. The tilt angle varied from − 60º to 60º with 3º increment to acquire 41 movies using SerialEM^39^, and the order of tilt series was as the following tilt angle in degree: (0, 3, 6, 9) -> (− 3, − 6, − 9) -> (12, 15, 18) -> (− 12, − 15, − 18) -> (21, 24, 27) -> (− 21, − 24, − 27) -> (30, 33, 36) -> (− 30, − 33, − 36) -> (39, 42, 45) -> (− 39, − 42, − 45, − 48, − 51, − 54, − 57, − 60) -> (48, 51, 54, 57, 60). Each movie was fractionated to 5 frames (0.1 s each, total exposure 0.5 s per tilt angle) with a total dose of 98 e^−^/Å^2^. For GroEL-Gh, the nominal magnification was 20,000×, corresponding to 2.37 Å/pixel. Defocus was set to − 3.0 μm. The tilt angle varied from − 60º to 60º with 3º increment to acquire 41 movies using SerialEM^39^, and the order of tilt series was as the following tilt angle in degree: (0, 3, 6) -> (− 3, − 6) -> (9, 12) -> (− 9, − 12) -> (15, 18) -> (− 15, − 18) -> (21, 24) -> (− 21, − 24) -> (27, 30) -> (− 27, − 30) -> (33, 36) -> (− 33, − 36) -> (39, 42) -> (− 39, − 42) -> (45, 48, 51, 54, 57, 60) -> (− 45, − 48, − 51, − 54, − 57, − 60). Each movie was fractionated to 5 frames (0.25 s each, total exposure 1.25 s per tilt angle) with a total dose of 40 e^−^/Å^2^. Tomograms were reconstructed using IMOD^50^. Images were aligned without using gold nanoparticles by patch tracking (for GroEL-EG) or by tracking ice contaminations as seeds (for GroEL-Gh).

## Supplementary Information

Supplementary Figures.

Supplementary Video 1.

Supplementary Video 2.

Supplementary Video 3.

## Data Availability

Density maps and raw multi-frame movies are available at EMDB and EMPIAR with accession codes EMD-31310 and EMPIAR-10948 (GroEL-EG), EMD-34743 and EMPIAR-11304 (GroEL-Q), EMD-31311 and EMPIAR-10949 (GroEL-Gl), EMD-32159 and EMPIAR-10954 (GroEL-Gh), EMD-34106 and EMPIAR-11170 (GroEL-EG3m), EMD-32160 and EMPIAR-10951 (SARS-CoV-2 Spike-EG), EMD-32161 and EMPIAR-10952 (SARS-CoV-2 Spike-Q), EMD-31312 (V_1_-ATPase-EG), EMD-32162 and EMPIAR-10955 (GAPDH-EG), EMD-31313 and EMPIAR-10950 (β-galactosidase-EG), and EMD-31314 and EMPIAR-10953 (apoferritin-EG), respectively.
