# Supplementary material for: Epoxidized graphene grid for highly efficient high-resolution cryoEM structural analysis
Source: Sci Rep. 2023 Feb 8;13:2279. doi: 10.1038/s41598-023-29396-0 (PMC9908306; doi:10.1038/s41598-023-29396-0)
Supplement: Supplementary file 1 — Supplementary Figures. [file 41598_2023_29396_MOESM1_ESM.docx]

**Supplementary Information for**

**Epoxidized graphene grid for highly efficient high-resolution cryoEM structural analysis**

**Junso Fujita^1,2,3^†, Fumiaki Makino^1,2,4^†, Haruyasu Asahara^3,5^†, Maiko Moriguchi^3^, Shota Kumano^3^, Itsuki Anzai^6^, Jun-ichi Kishikawa^7,8^, Yoshiharu Matsuura^9,10^, Takayuki Kato^7^, Keiichi Namba^1,2,11,^*, Tsuyoshi Inoue^3,5,12,^***

^1^Graduate School of Frontier Biosciences, Osaka University, 1-3 Yamadaoka, Suita, Osaka 565-0871, Japan.

^2^JEOL YOKOGUSHI Research Alliance Laboratories, Osaka University, 1-3 Yamadaoka, Suita, Osaka 565-0871, Japan.

^3^Graduate School of Pharmaceutical Sciences, Osaka University, 1-6 Yamadaoka, Suita, Osaka 565-0871, Japan.

^4^JEOL Ltd, 3-2-1 Musashino, Akishima, Tokyo 196-8558, Japan.

^5^Open and Transdisciplinary Research Initiatives, Osaka University, 2-8 Yamadaoka, Suita, Osaka 565-0871, Japan.

^6^Department of Molecular Virology, Research Institute for Microbial Diseases, Osaka University, 3-1 Yamadaoka, Suita, Osaka 565-0871, Japan.

^7^Institute for Protein Research, Osaka University, 3-2 Yamadaoka, Suita, Osaka, 565-0871, Japan.

^8^Department of Molecular Biosciences, Kyoto Sangyo University, Motoyama Kamigamo, Kita-ku, Kyoto, 603-8555, Japan.

^9^Center for Infectious Disease Education and Research, Osaka University, 2-8 Yamadaoka, Suita, Osaka 565-0871, Japan.

^10^Laboratory of Virus Control, Research Institute for Microbial Diseases, Osaka University, 3-1 Yamadaoka, Suita, Osaka 565-0871, Japan.

^11^RIKEN Center for Biosystems Dynamics Research and SPring-8 Center, 1-3 Yamadaoka, Suita, Osaka 565-0871, Japan.

^12^dotAqua Inc., 2-1 Yamadaoka, Suita, Osaka, Japan.

*Correspondence to: Tsuyoshi Inoue (t_inoue@phs.osaka-u.ac.jp) or Keiichi Namba ([keiichi@fbs.osaka-u.ac.jp](mailto:keiichi@fbs.osaka-u.ac.jp))

†These authors are equally contributed to this work.


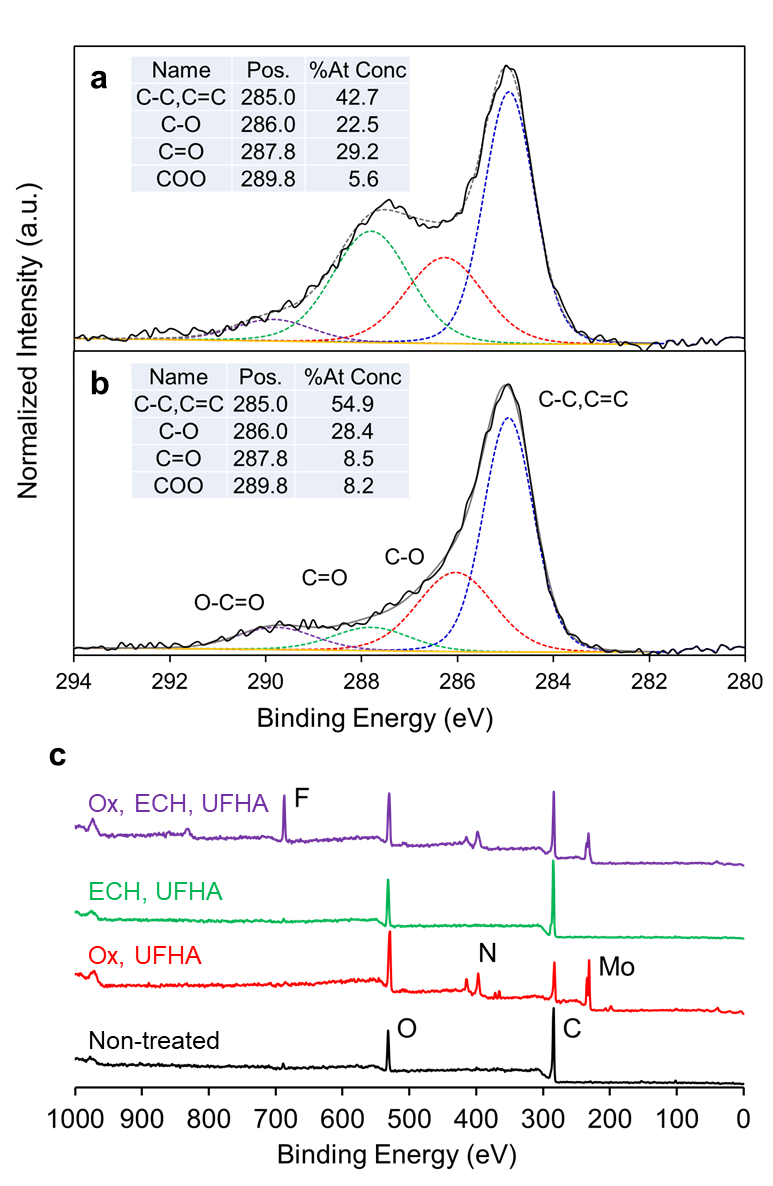


Supplementary Figure 1. XPS spectra of chemically modified graphene. (a–b) C 1s high-resolution spectra of plasma-treated (a) and ClO_2_^•^-treated (b) graphene on a silicon wafer. The inset shows binding energies of the C 1s electrons in the functional groups that were used in the fitting procedure and the composition ratios. (c) XPS spectra of graphene (black line), UFHA-treated oxidized graphene (red line), epichlorohydrin- and UFHA-treated graphene (green line), on the Quantifoil Mo grid, and UFHA-treated EG-grid (purple line).


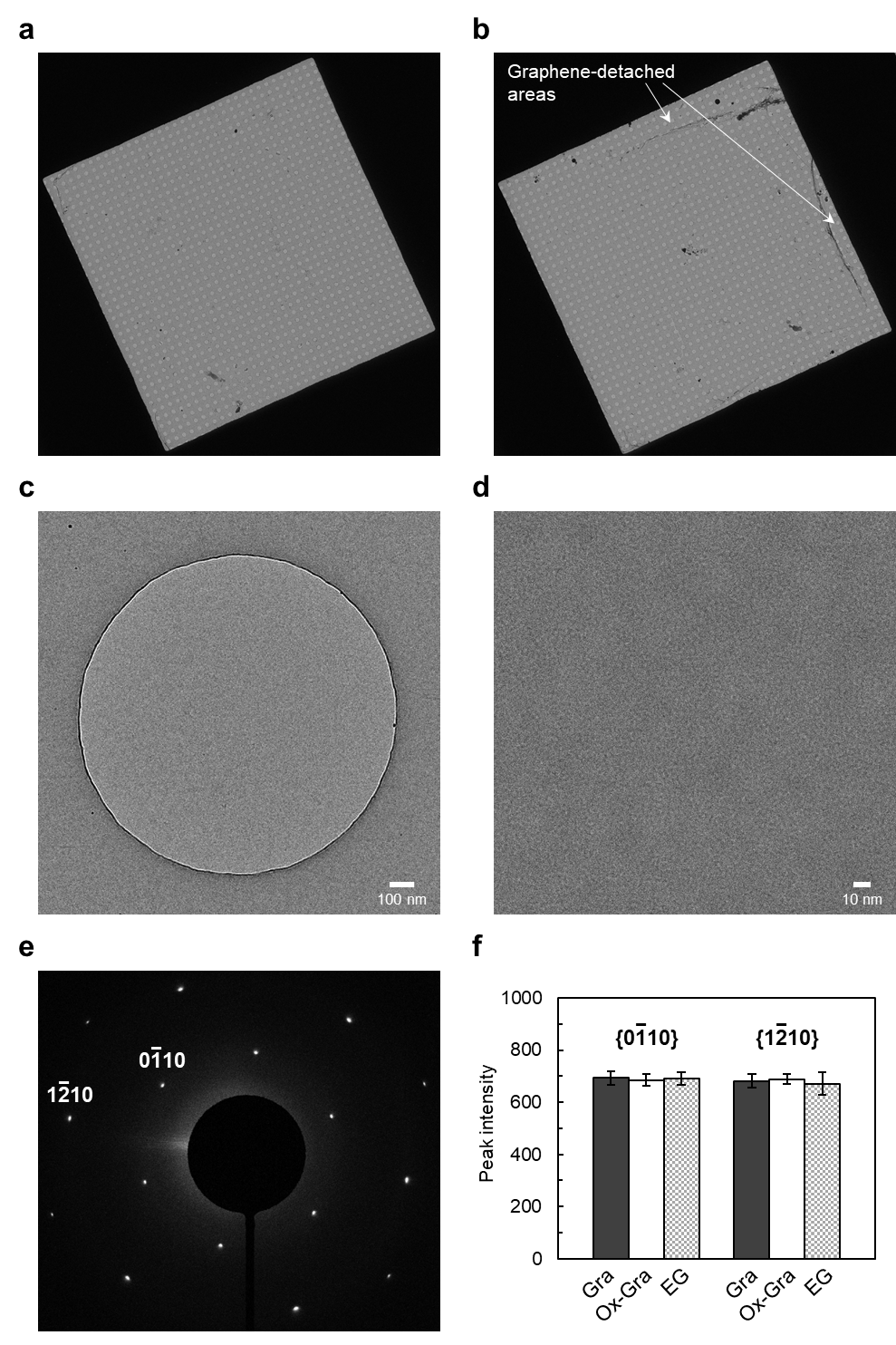


Supplementary Figure 2. CryoEM images of a typical EG-grid to show its quality. (a–b) Images of two different squares of an EG-grid: one with graphene fully covered (a) and the other with graphene partially detached (b). (c) Image of a single hole of 1.2 mm diameter with suspended graphene at a magnification of 8,000x. (d) Image of the same hole as shown in (c) at a higher magnification of 60,000x. (e) Electron diffraction pattern from graphene in the hole shown in (c). (f) Comparison of diffraction peak intensities from suspended graphene on three different types of grids: non-oxidized graphene grid (Gra); ClO_2_^•^ oxidized graphene grid (Ox-Gra); and ClO_2_^•^ oxidized and epoxidized graphene grid (EG). The peak intensities of diffraction spots are shown as the mean ± standard deviation of the mean (n = 30: 6 equivalent diffraction spots per one area x 5 areas in each grid).


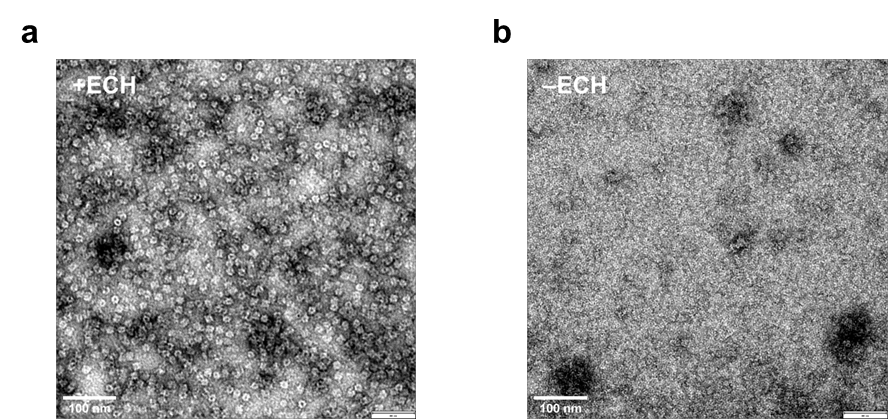


Supplementary Figure 3. Protein binding activity of the EG-grid. (a–b) Typical electron micrographs (30,000 x) of GroEL negatively stained (a) on an EG-grid after washing by GroEL buffer three times before staining and (b) on a grid prepared in the same way as (a) except omitting the epoxidation process by ECH.


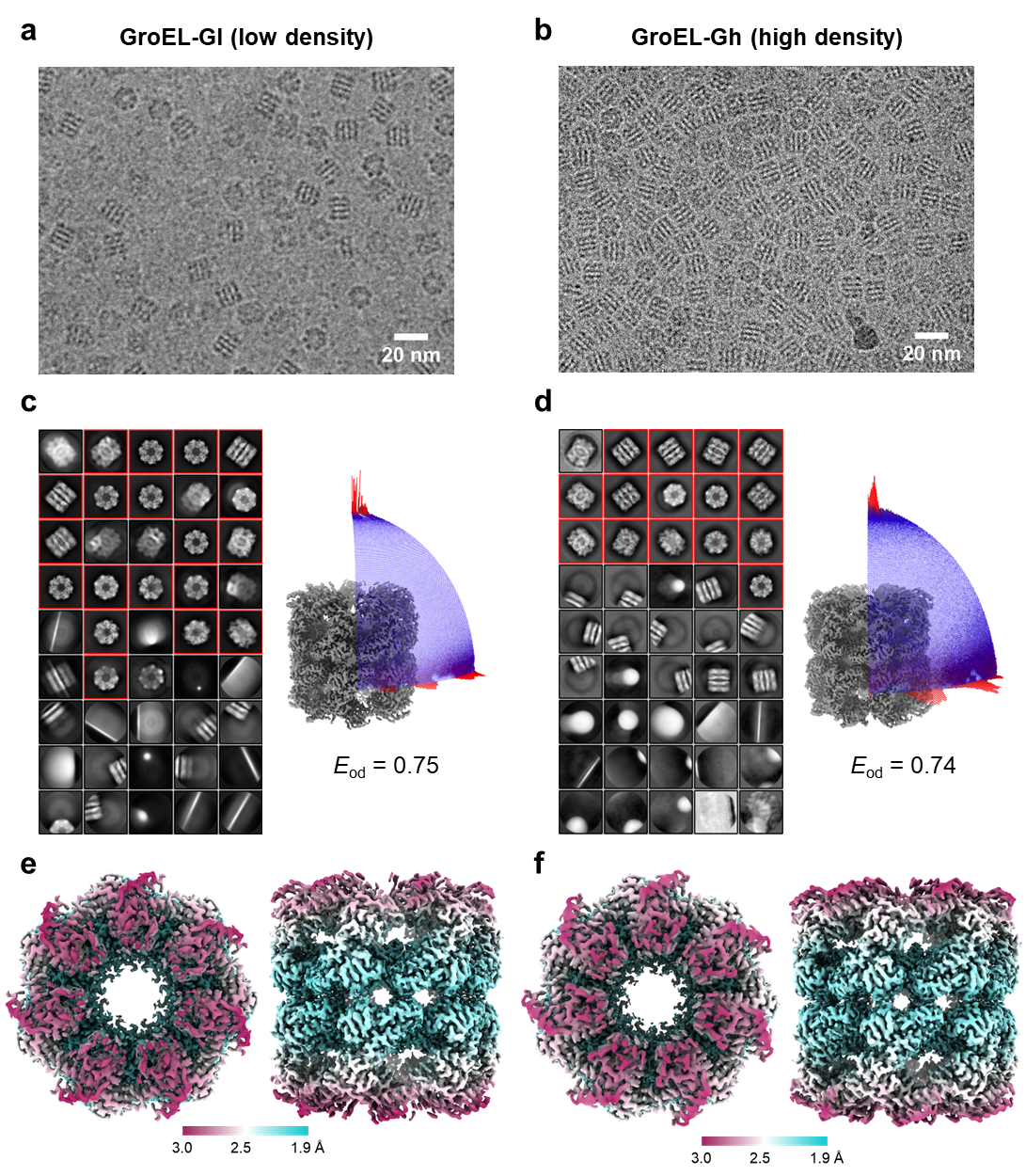


Supplementary Figure 4. CryoEM image analyses of the GroEL on glow discharged graphene grids. (a–b) Typical cryoEM images of GroEL packed with low density (GroEL-Gl) (a) and high density (GroEL-Gh) (b) on the different glow discharged graphene grids. (c–d) 2D class averages from the GroEL-Gl (c) and GroEL-Gh (d) dataset. The class averages are aligned in descending order of particle numbers from left to right and top to bottom. The classes selected for the following analysis are indicated with red boxes. Right panel shows angular distribution of particles used in the final refinement for each dataset. Final 3D maps are shown for reference. The efficiencies (*E*_od_) calculated with cryoEF are also shown. (e–f) Final 3D maps of GroEL-Gl (e) and GroEL-Gh (f) dataset in two orthogonal views: top view, left panels; and side view, right panels. The local resolution distributions are colored as in the color bars.


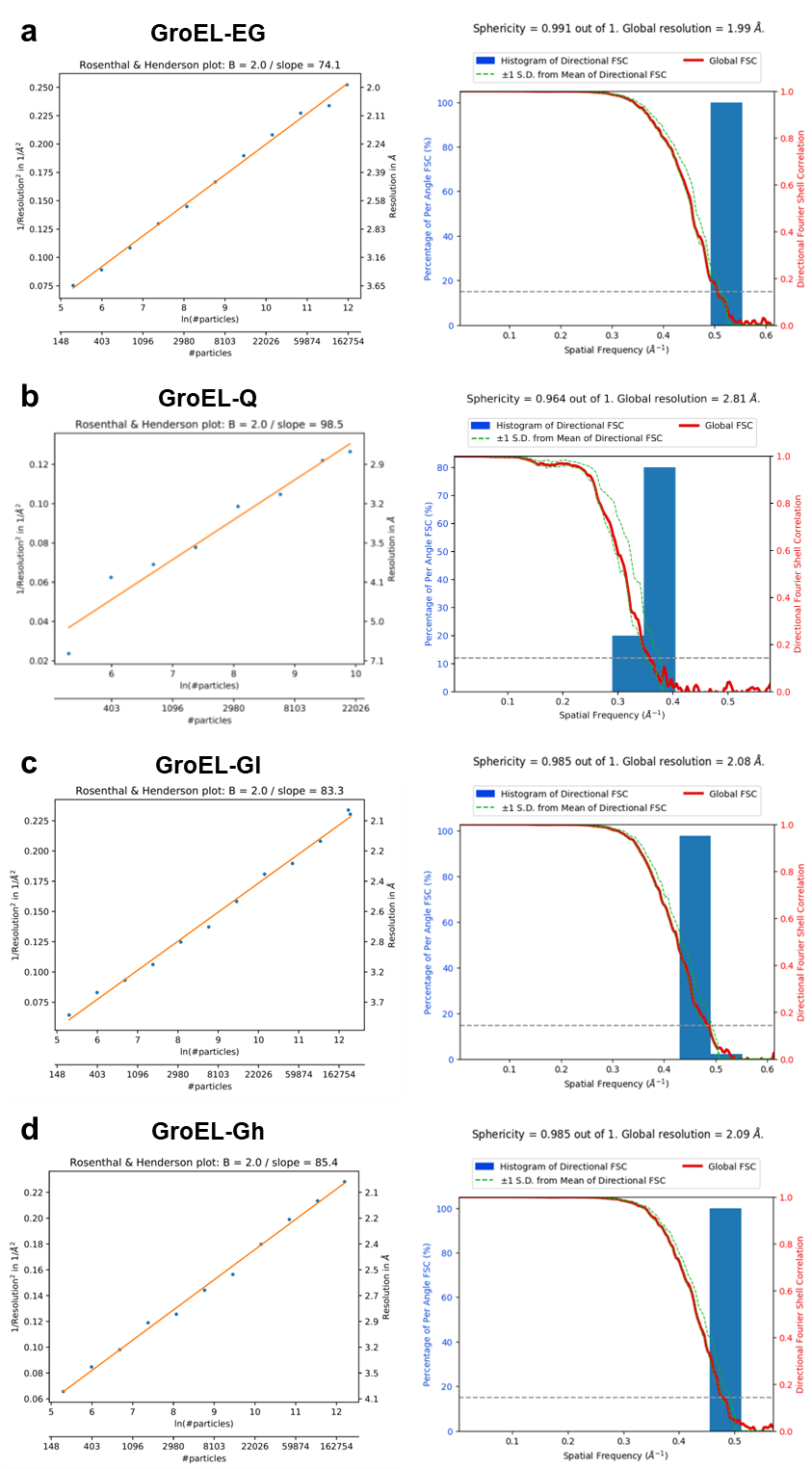


Supplementary Figure 5. CryoEM image analyses of the GroEL. (a–d) Rosenthal-Henderson B-factor plots (left panels) and FSC curves and sphericities calculated by the 3DFSC server (https://3dfsc.salk.edu) for the final maps (right panels) of the GroEL-EG (a), GroEL-Q (b), GroEL-Gl (c), and GroEL-Gh (d) dataset. The dashed line indicates the FSC = 0.143 criterion.


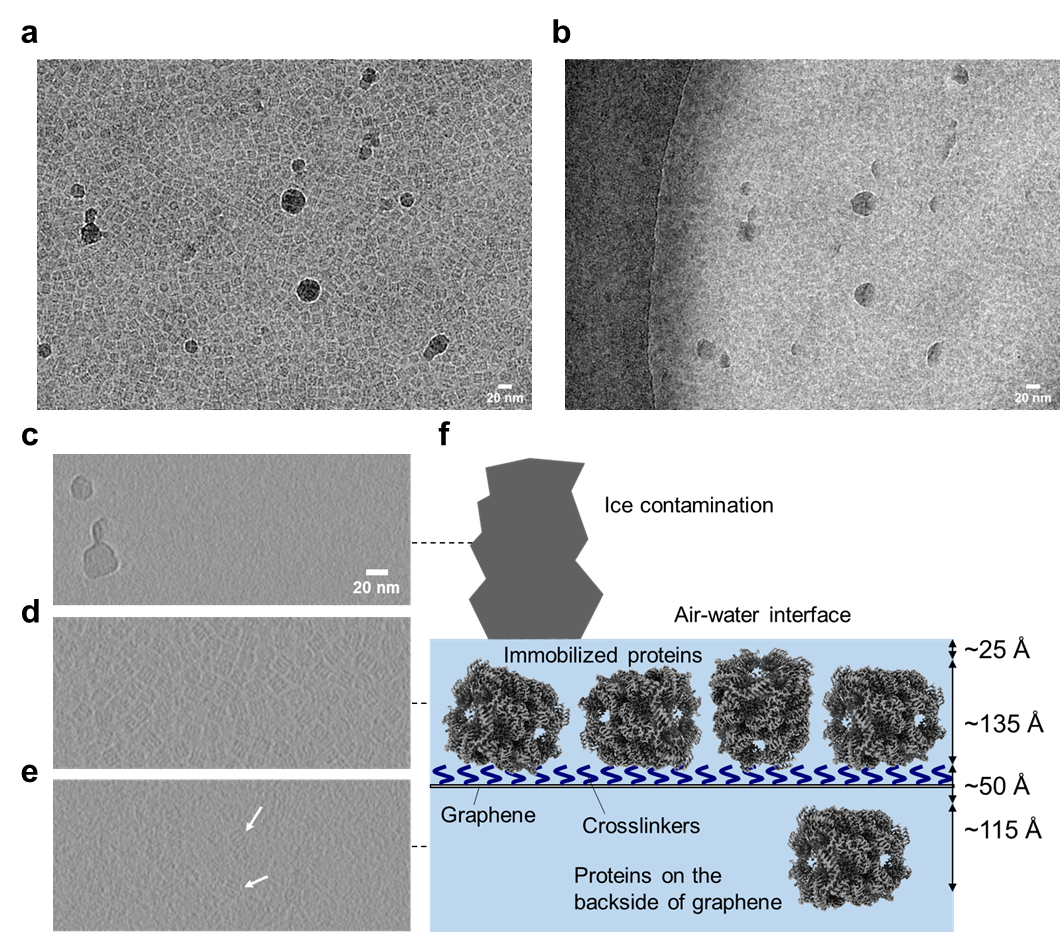


Supplementary Figure 6. Cryo-ET of GroEL-embedded ice film on the EG-grid. (a–b) CryoEM images of GroEL-embedded ice on the EG-grid at a tilt angle of 0º (a) and –54º (b). (c–e) Three-layer images extracted from the tomogram reconstructed from the area corresponding to those shown in (a) and (b). (c) A layer near the surface of vitreous ice where the same pair of ice contaminants seen in the left part of (a) and (b) can be seen. (d) A vitreous ice layer containing GroEL particles, which is ~240 Å lower than that of (c). (e) Another layer showing a few GroEL particles (white arrows), which is ~225 Å lower than that of (d). See the whole tomogram in Supplementary Movie 2. (f) Schematic illustration of ice-embedded GroEL particles attached on the graphene surface of the EG-grid.


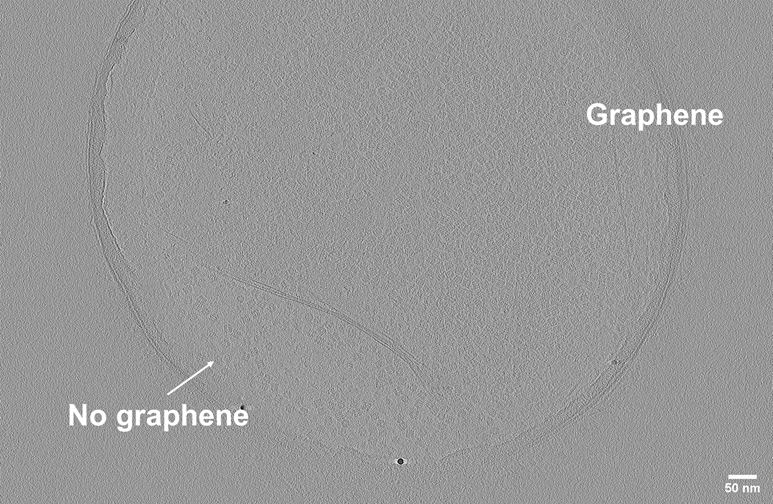


Supplementary Figure 7. Cryo-ET of GroEL-embedded ice film on the glow discharged graphene grid. A layer image extracted from the reconstructed tomogram. The hole included both area with and without graphene. See the whole tomogram in Supplementary Movie 3.


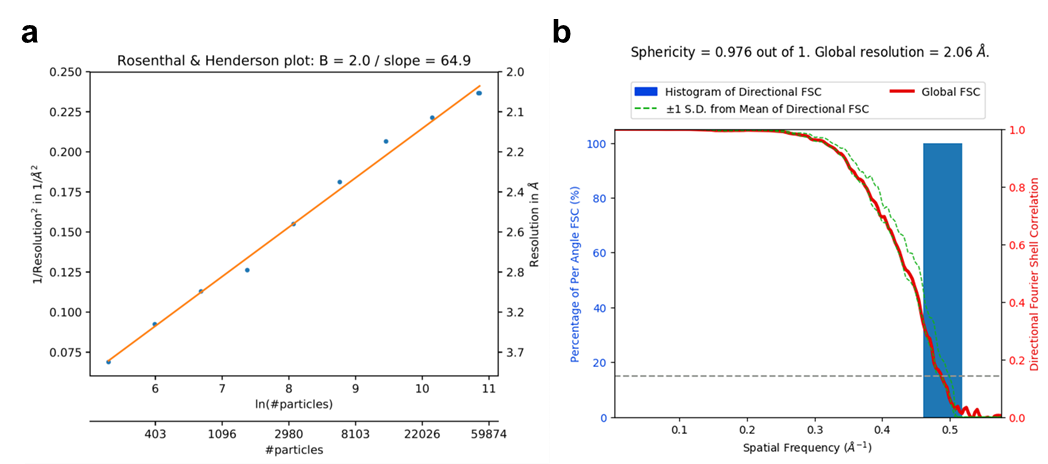


Supplementary Figure 8. CryoEM image analysis of the GroEL-EG3m dataset. (a) Rosenthal-Henderson B-factor plot. (b) FSC curve and sphericity calculated by the 3DFSC server (https://3dfsc.salk.edu) for the final map. The dashed line indicates the FSC = 0.143 criterion.


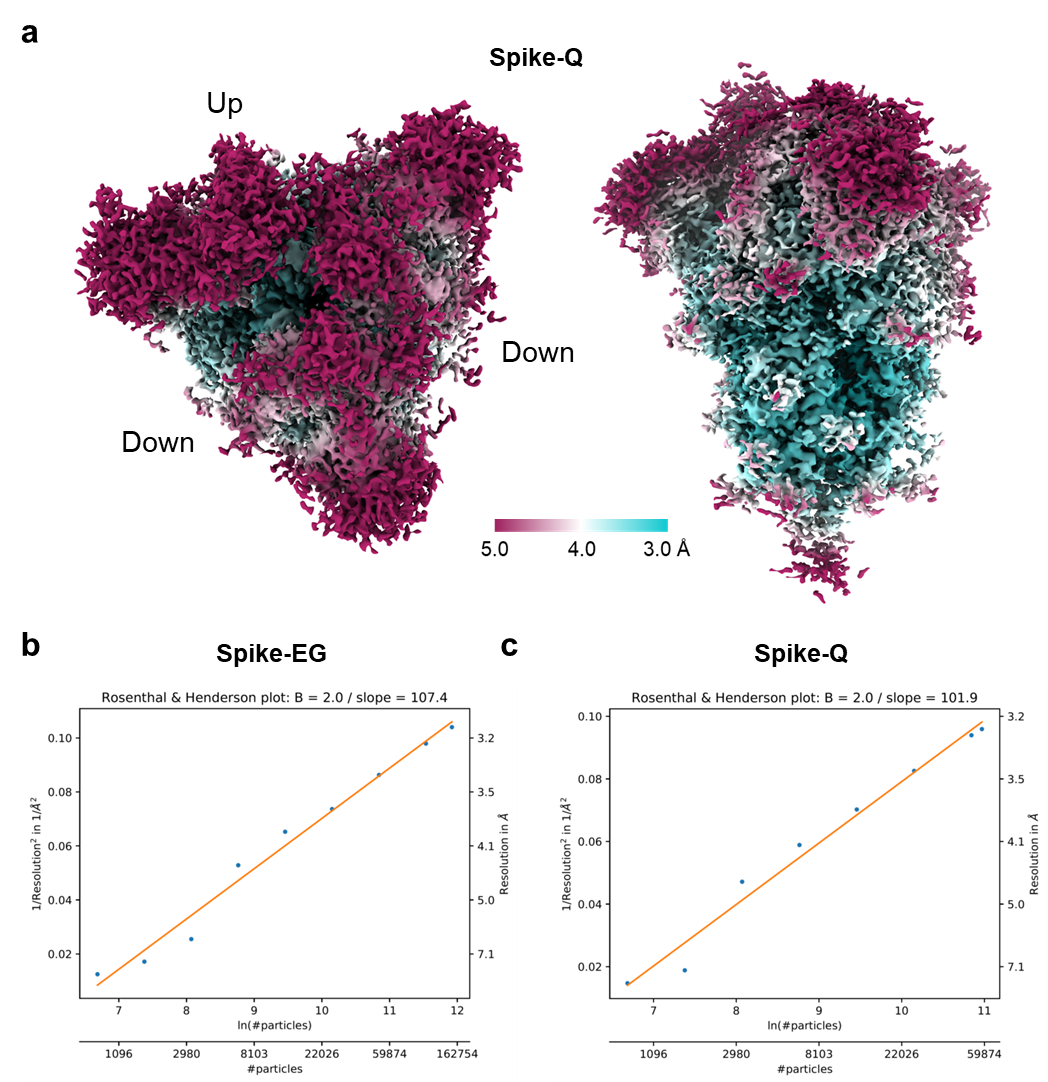


Supplementary Figure 9. CryoEM image analysis of the Spike-Q dataset. (a) Final 3D map of the spike-Q dataset in two orthogonal views: top view, left panel; and side view, right panel. The local resolution distribution is colored as in the color bar. (b–c) Rosenthal-Henderson B-factor plots of the Spike-EG (b) and Spike-Q (c) dataset.


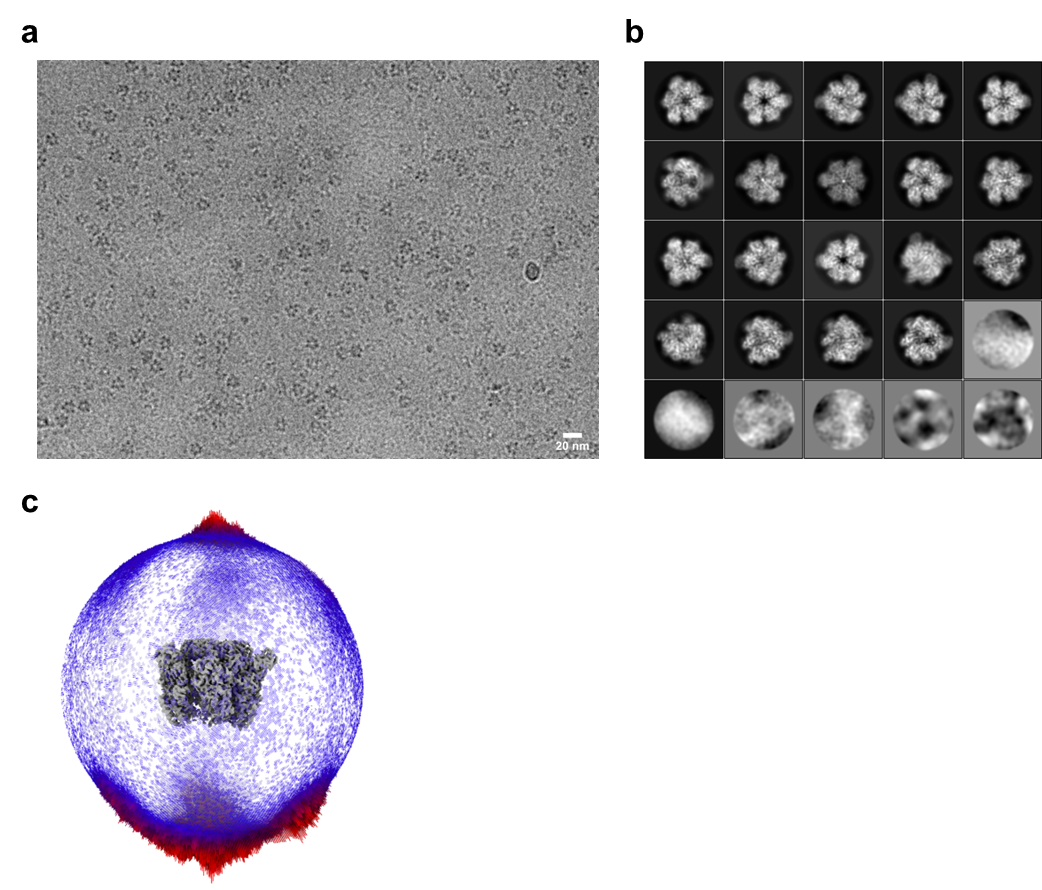


Supplementary Figure 10. CryoEM image analysis of the V_1_-ATPase-EG dataset. (a) Typical cryoEM image (50,000 x) of the A_3_B_3_ ring of V_1_-ATPase on the EG-grid. (b) Top 25 2D class averages aligned in the descending order of particle numbers from left to right and top to bottom. (c) Angular distribution of particles used in the final refinement. The final 3D map is also shown in grey for reference.


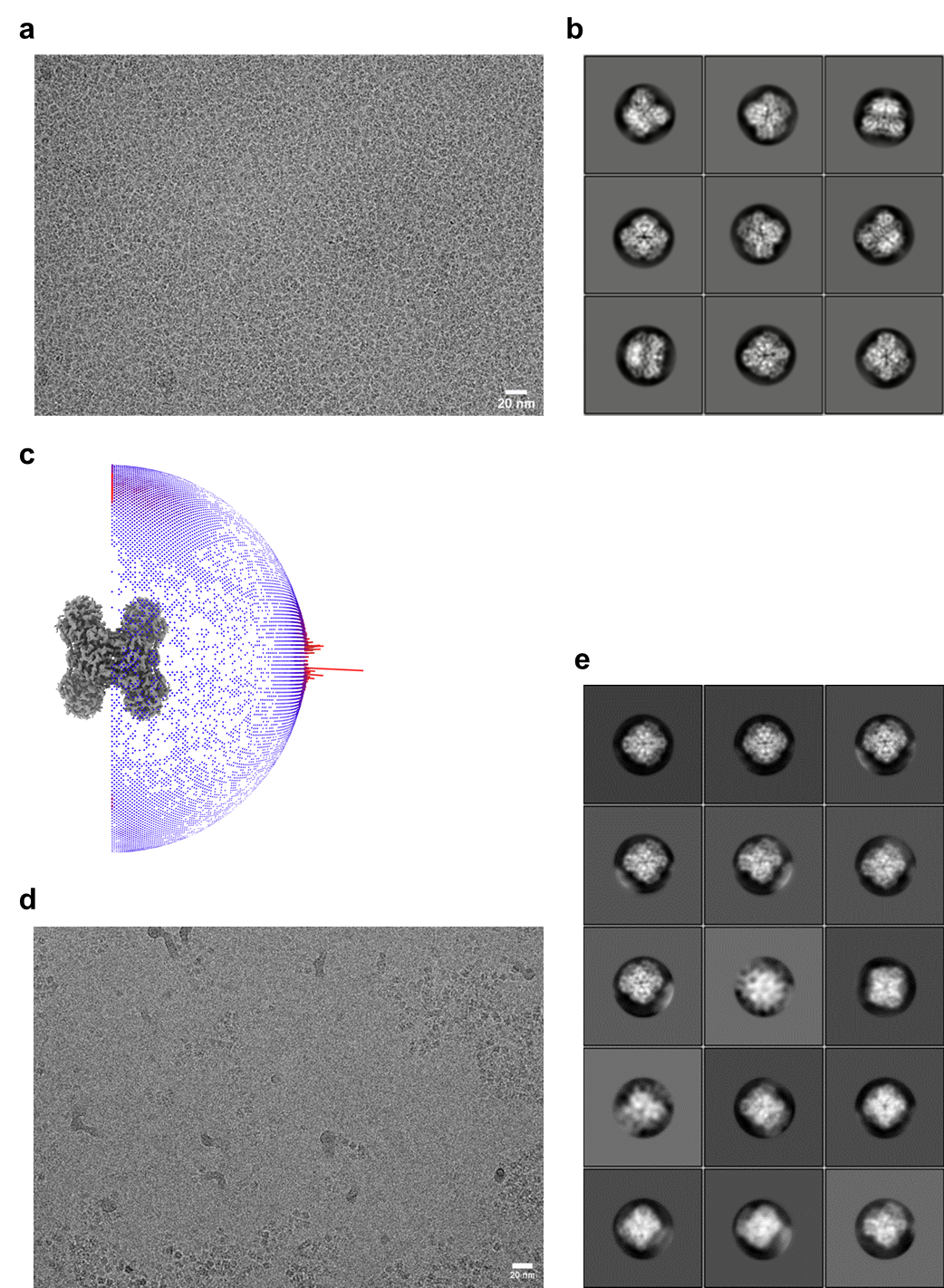


Supplementary Figure 11. CryoEM image analyses of the GAPDH-EG and GAPDH-Q dataset. (a) Typical cryoEM image (60,000 x) of GAPDH on the EG-grid. (b) Top 9 2D class averages of GAPDH-EG aligned in the descending order of particle numbers from left to right and top to bottom. (c) Angular distribution of particles of GAPDH-EG used in the final refinement. The final 3D map is also shown in grey for reference. (d) Typical cryoEM image (60,000 x) of GAPDH on the Quantifoil grid. (e) Top 15 2D class averages of GAPDH-Q aligned in the descending order of particle numbers from left to right and top to bottom.


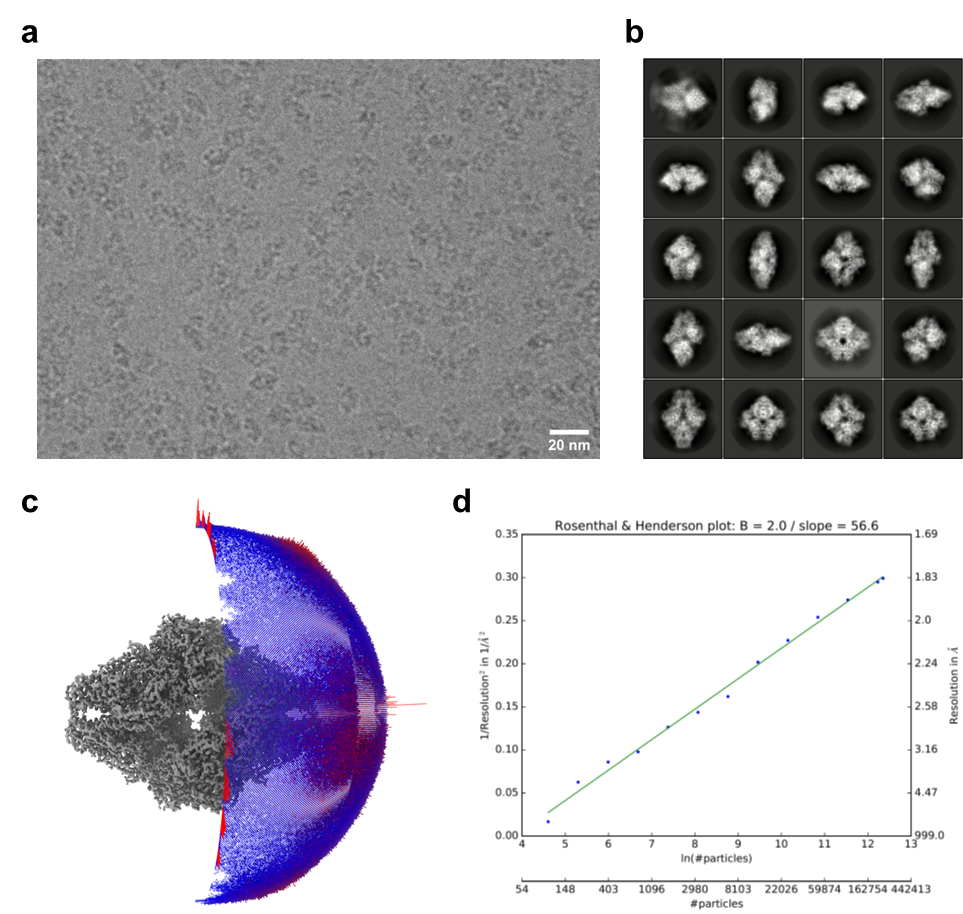


Supplementary Figure 12. CryoEM image analysis of the β-galactosidase-EG dataset. (a) Typical cryoEM image (100,000 x) of β-galactosidase on the EG-grid. (b) Top 20 2D class averages aligned in the descending order of particle numbers from left to right and top to bottom. (c) Angular distribution of particles used in the final refinement. The final 3D map is also shown in grey for reference. (d) Rosenthal-Henderson B-factor plot.


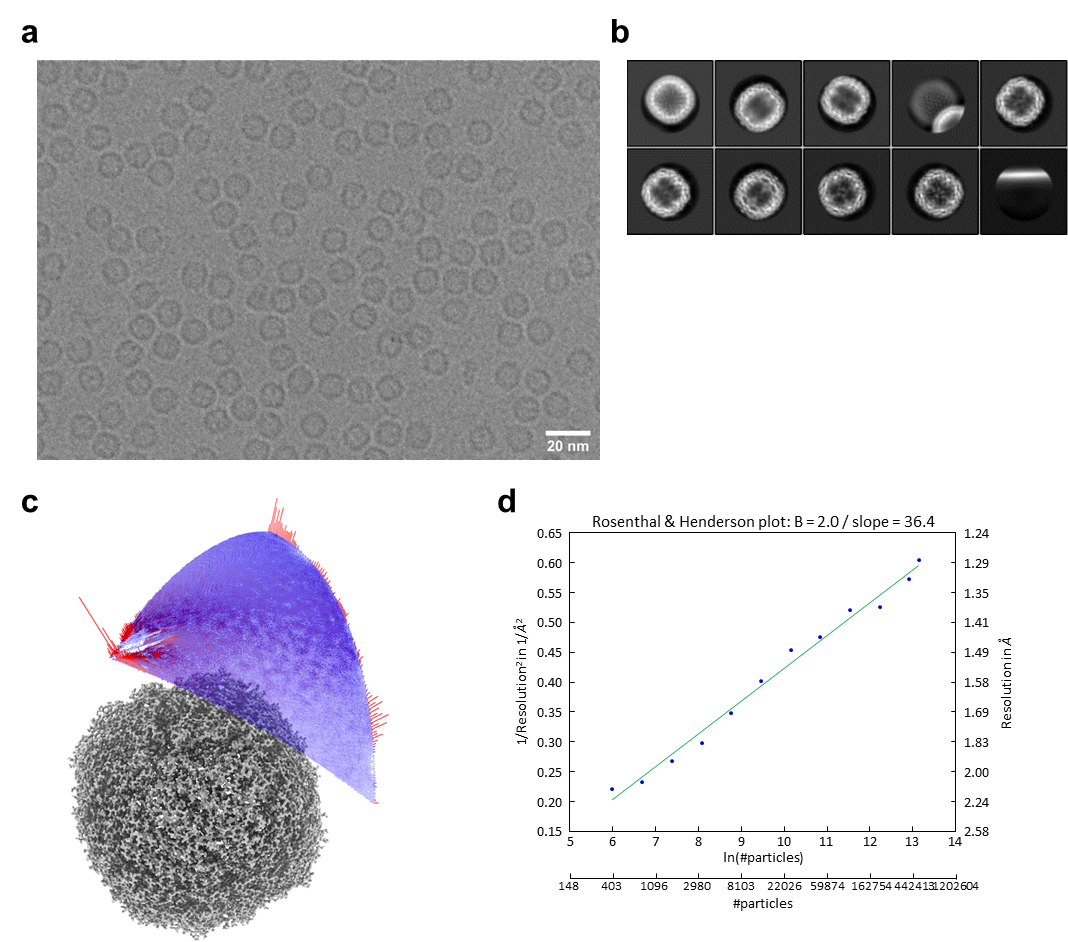


Supplementary Figure 13. CryoEM image analysis of the apoferritin-EG dataset. (a) Typical cryoEM image (120,000 x) of apoferritin on the EG-grid. (b) Top 10 2D class averages aligned in the descending order of particle numbers from left to right and top to bottom. (c) Angular distribution of particles used in the final refinement. The final 3D map is also shown in grey for reference. (d) Rosenthal-Henderson B-factor plot.


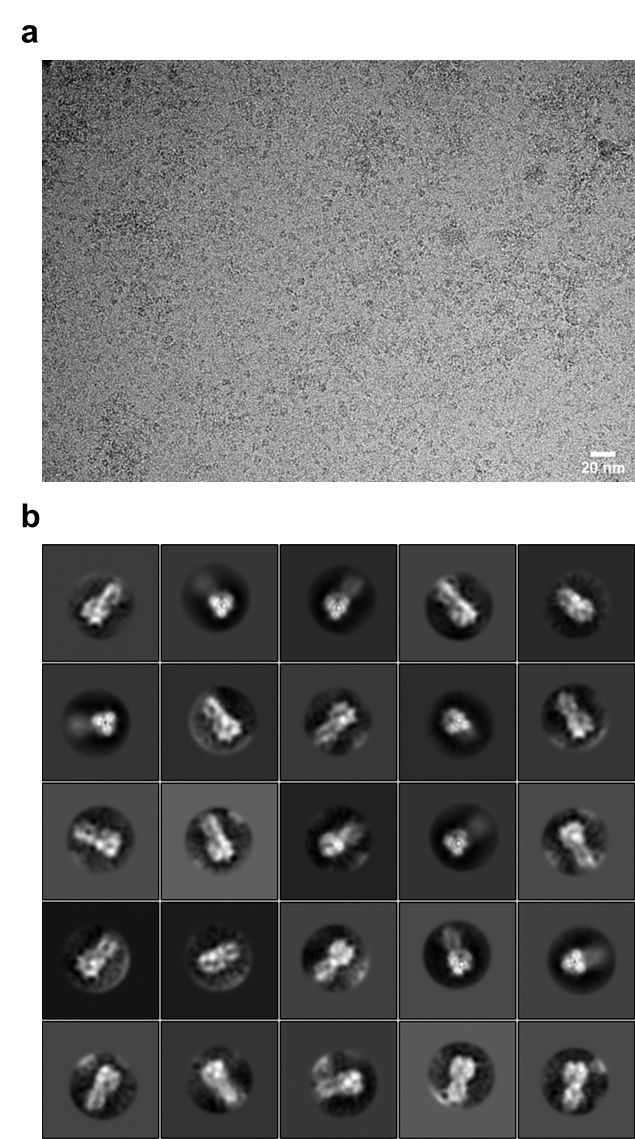


Supplementary Figure 14. CryoEM image analysis of the hemagglutinin on EG-grid. (a) Typical cryoEM image (60,000 x) of hemagglutinin on the EG-grid. (b) Top 25 2D class averages aligned in the descending order of particle numbers from left to right and top to bottom.

Supplementary Table 1. CryoEM data collection and image processing.

| Dataset | GroEL-EG | GroEL-Q | GroEL-Gl | GroEL-Gh |
| --- | --- | --- | --- | --- |
| EMDB accession code | EMD-31310 | EMD-34743 | EMD-31311 | EMD-32159 |
| EMPIAR accession code | 10948 | 11304 | 10949 | 10954 |
| Magnification | 60,000 | 60,000 | 60,000 | 60,000 |
| Voltage (kV) | 300 | 300 | 300 | 300 |
| Electron exposure (e^−^/Å^2^) | 40 | 40 | 40 | 40 |
| Defocus range (μm) | −0.5 to −2.0 | −0.5 to −2.0 | −0.5 to −2.0 | −0.5 to −2.0 |
| Pixel size (Å) | 0.813 | 0.867 | 0.816 | 0.878 |
| Symmetry imposed | D7 | D7 | D7 | D7 |
| Initial images (no.) | 504 | 553 | 5,614 | 649 |
| Optics group (no.) | 10 | 6 | 11 | 13 |
| Initial particle images (no.) | 178,215 | 39,492 | 1,118,169 | 340,446 |
| Final particle images (no.) | 158,485 | 20,061 | 215,011 | 198,677 |
| Final/Initial particle ratio (%) | 88.9 | 50.8 | 19.2 | 58.4 |
| Average of final particles  per image (no.) | 314.5 | 36.3 | 38.3 | 306.1 |
| Map resolution (Å)  FSC threshold | 1.99  0.143 | 2.81  0.143 | 2.08  0.143 | 2.09  0.143 |
| Map resolution range (Å) | 1.93–3.04 | 2.65–4.36 | 2.02–3.09 | 2.06–3.07 |

| Dataset | GroEL-EG3m | SARS-CoV-2  Spike-EG | SARS-CoV-2  Spike-Q | V_1_-ATPase-EG |
| --- | --- | --- | --- | --- |
| EMDB accession code | EMD-34106 | EMD-32160 | EMD-32161 | EMD-31312 |
| EMPIAR accession code | 11170 | 10951 | 10952 | − |
| Magnification | 60,000 | 60,000 | 60,000 | 50,000 |
| Voltage (kV) | 300 | 300 | 300 | 300 |
| Electron exposure (e^−^/Å^2^) | 40 | 60 | 60 | 62 |
| Defocus range (μm) | −0.5 to −2.0 | −0.5 to −2.0 | −0.5 to −2.0 | −1.0 to −2.5 |
| Pixel size (Å) | 0.868 | 0.870 | 0.870 | 0.990 |
| Symmetry imposed | D7 | C1 | C1 | C1 |
| Initial images (no.) | 500 | 1,163 | 1,029 | 2,052 |
| Optics group (no.) | 10 | 12 | 10 | 11 |
| Initial particle images (no.) | 154,796 | 602,489 | 244,439 | 1,193,032 |
| Final particle images (no.) | 52,043 | 150,316 | 58,102 | 238,765 |
| Final/Initial particle ratio (%) | 33.6 | 24.9 | 23.8 | 20.0 |
| Average of final particles  per image (no.) | 104.1 | 129.2 | 56.5 | 116.4 |
| Map resolution (Å)  FSC threshold | 2.06  0.143 | 3.10  0.143 | 3.23  0.143 | 3.03  0.143 |
| Map resolution range (Å) | 2.03–3.30 | 2.82–14.7 | 2.95–12.2 | 2.85–4.66 |

Supplementary Table 1. (Continued from previous page)

| Dataset | GAPDH-EG | β-gal-EG | Apoferritin-EG |
| --- | --- | --- | --- |
| EMDB accession code | 32162 | 31313 | 31314 |
| EMPIAR accession code | 10955 | 10950 | 10953 |
| Magnification | 60,000 | 100,000 | 120,000 |
| Voltage (kV) | 300 | 300 | 300 |
| Electron exposure (e^−^/Å^2^) | 40 | 40 | 40 |
| Defocus range (μm) | −0.5 to −2.0 | −0.5 to −2.0 | −0.3 to −1.3 |
| Pixel size (Å) | 0.813 | 0.495 | 0.490 |
| Symmetry imposed | D2 | D2 | O |
| Initial images (no.) | 241 | 3,242 | 7,500 |
| Optics group (no.) | 8 | 16 | 12 |
| Initial particle images (no.) | 301,451 | 491,997 | 793,398 |
| Final particle images (no.) | 88,731 | 231,395 | 527,261 |
| Final/Initial particle ratio (%) | 29.4 | 47.0 | 66.5 |
| Average of final particles  per image (no.) | 368.2 | 71.4 | 70.3 |
| Map resolution (Å)  FSC threshold | 2.16  0.143 | 1.81  0.143 | 1.29  0.143 |
| Map resolution range (Å) | 2.10–2.98 | 1.76–2.35 | 1.28–1.47 |

Supplementary Movie 1. Preparation of the EG-grid.

Supplementary Movie 2. Reconstructed tomogram of GroEL on the EG-grid. The section starts from near the surface of vitreous ice, goes through the GroEL and graphene layer on the EG-grid and then goes backward. Tomogram thickness is 100 nm.

Supplementary Movie 3. Reconstructed tomogram of GroEL on the glow discharged graphene grid. The section starts from near the surface of vitreous ice, goes through the GroEL and graphene layer and then goes backward. Tomogram thickness is 100 nm.
